# Luminescent Properties and Cytotoxic Activity of 2-phenylbenzoxazole Fluorosulfate Derivatives

**DOI:** 10.3390/ijms26157261

**Published:** 2025-07-27

**Authors:** Nadezhda V. Danilenko, Mariia O. Lutsuk, Alexey A. Ryadun, Dmitry I. Pavlov, Evgenii V. Plotnikov, Daria D. Eskova, Yulia D. Klimenko, Andrei S. Potapov, Andrei I. Khlebnikov

**Affiliations:** 1 Kizhner Research Center, Tomsk Polytechnic University, 634050 Tomsk, Russia; nadezhda.dani@gmail.com (N.V.D.); lutsukma@gmail.com (M.O.L.); aikhl@chem.org.ru (A.I.K.); 2 Nikolaev Institute of Inorganic Chemistry, Siberian Branch of the Russian Academy of Sciences, 3 Lavrentiev Ave., 630090 Novosibirsk, Russia; ryadunalexey@mail.ru (A.A.R.); pavlov@niic.nsc.ru (D.I.P.);; 3 Research School of Chemistry and Applied Biomedical Sciences, Tomsk Polytechnic University, 30 Lenin Avenue, 634034 Tomsk, Russia; dde5@tpu.ru (D.D.E.); yuliaklim1207@mail.ru (Y.D.K.); 4 Department of Chemistry, Siberian State Medical University, 2 Moscow Trakt, 634050 Tomsk, Russia

**Keywords:** benzoxazole, SuFEx, fluorescence, antitumor activity

## Abstract

The synthesis of 2-phenylbenzoxazole fluorosulfate derivatives was carried out using the SuFEx reaction. To study the anticancer properties of the obtained compounds, the cell lines PC-3 (obtained from prostate adenocarcinoma), BT-474, and MCF-7 (both obtained from breast carcinoma) were used. The cytotoxicity on murine 3T3L1 embryonic was also investigated. Among the tested compounds, the *ortho*-substituted fluorosulfate derivative (**BOSo**) exhibited significant cytotoxicity against MCF-7 cells. The biological findings are consistent with molecular docking results, which revealed a structural similarity between **BOSo** and known inhibitors of hER and HER2 receptors—tamoxifen and SYR127063. Therefore, **BOSo** shows promise as a potential therapeutic agent with antiproliferative properties. The photoluminescent characteristics of the fluorosulfate derivatives were examined in the solid state, in acetonitrile solution and in PBS, with the highest quantum yields reaching up to 64% for the *para*-fluorosulfate derivative in acetonitrile. Overall, these compounds demonstrate considerable potential for the development of new multifunctional molecular tools that combine biological activity with fluorescent properties.

## 1. Introduction

**Figure 1:**
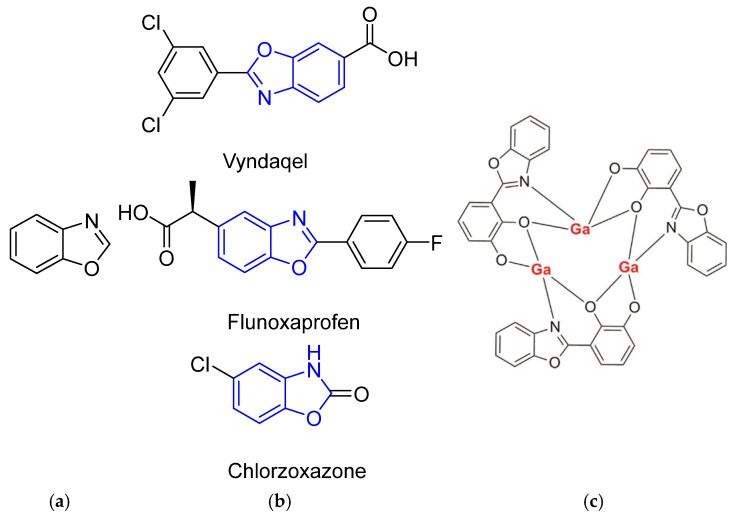
(**a**) Structure of benzoxazole; (**b**) drug molecules with benzoxazole heterocycle; (**c**) one of possible Ga^3+^ coordination compounds used for bioimaging [9].

The development of novel benzoxazole derivatives continues to attract significant research interest due to several important factors. Firstly, compounds containing benzoxazole moiety (Figure 1a) in their molecular structure demonstrate broad biological activities. Such an important class of heterocyclic compounds exhibited antimicrobial [1], antiviral [2], anticancer activity [3], inhibitory activity on topoisomerase II [4], and is used in medicine (Figure 1b).

On the other hand, the 2-phenylbenzoxazole fragment is extensively utilized as a fundamental substructure across various fields, such as the development of pH and metal cation indicators [5,6], electro-optical materials [7], excited-state intramolecular proton transfer (ESIPT) dyes, and metal chelates (Figure 1c) for bioimaging [8,9] and electroluminescent devices [10]. 2-Phenylbenzoxazole derivatives are typically very stable and frequently demonstrate outstanding fluorescence efficiency [11], both in solution and in solid state [12,13].

The sulfur(VI) fluoride exchange (SuFEx)-mediated introduction of the fluorosulfate group in the 2-phenylbenzoxazole moiety can tune both biological and luminescent characteristics. A large number of studies were devoted to the use of the SuFEx reaction in drug development and biochemistry. For example, compounds containing the SO_2_F group demonstrated significant efficacy in enhancing memory function in Alzheimer’s disease patients [14]. Liu et al. [15] reported that structural modifications based on SuFEx chemistry substantially enhanced the anticancer activity of several NIH-approved therapeutic agents. SuFEx reaction enables the synthesis of various molecular probes, including fluorescent markers [16], while the fluorosulfate functional group facilitates efficient surface immobilization of target molecules [17].

In our previous work, we obtained the first fluorosulfate derivatives of 2-phenylbenzoxazole [18,19]; however, their biological and luminescent properties were not thoroughly studied. Here we report the synthesis and investigation of the previously unknown 2-(3-(fluorosulfato)phenyl)benzoxazole that completes this series of compounds.

## 2. Results and Discussion

### 2.1. Synthesis of Benzoxazole Derivatives

**Scheme 1:**
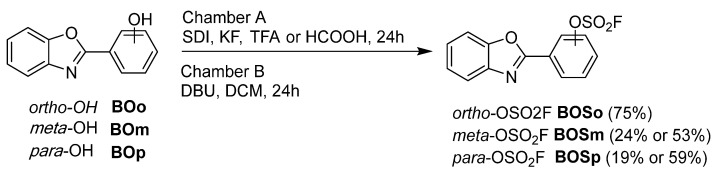
Synthesis of 2-phenylbenzoxazole fluorosulfates.

To expand the range of available benzoxazoles, we preliminarily synthesized their precursors, 2-(hydroxyphenyl)benzoxazoles, using the condensation of *o*-aminophenol with hydroxybenzoic acids [20]. Furthermore, with the use of the SuFEx reaction, we obtained 2-phenylbenzoxazole fluorosulfates) [18,19] (Scheme 1).

The reactions were conducted in the two-chamber reactor (Figure S1) following the principle described in paper [21]. Briefly, in chamber A, the interaction of 1,1′-sulfonyldiimidazole (SDI) with KF and liquid organic acid produced gaseous SO_2_F_2_, while chamber B was the zone of the click reaction.

In [21], optimal conditions for generating sulfuryl fluoride gas were identified to ensure higher yields of fluorosulfates. However, during the synthesis of **BOSo** [18] and **BOSp** [19], we encountered the problem of forming substantial amounts of by-products—*bis*(2-(benzoxazol-2-yl)phenyl)sulfate (**diBOSo**) and *bis*(4-(benzoxazol-2-yl)phenyl)sulfate (**diBOSp**)—due to further interaction of the target fluorosulfate with the starting compound **BOo**. It was shown [18] that increasing the excess of SO_2_F_2_ gas by 1.5 times nearly doubled the yield of the target product, with a 1.5-fold reduction in the yield of the by-product. In the present work, we have investigated the effect of increasing the excess of gaseous SO_2_F_2_ on the yields of fluorosulfates **BOSm** and **BOSp**. Additionally, we have studied the use of two different acids (CF_3_COOH and HCOOH) for the generation of SO_2_F_2_ in Chamber A (Scheme 1, Table 1).

Our results showed that the optimal ratio of 1,1′-sulfonyldiiimidazole (SDI) to KF is 2.5:6 [18], and the use of formic acid is preferred.

**Scheme 2:**
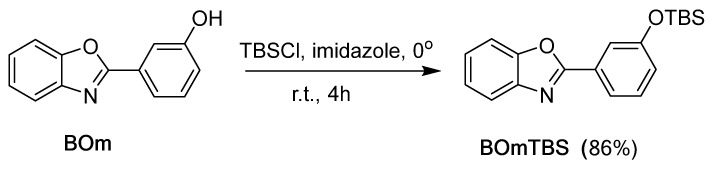
Synthesis of silyl ether derivatives of benzoxazole.

Pre-silylation of the hydroxyl group usually has a favorable effect on the SuFEx reaction [22], as silicon and fluorine atoms form a strong single bond (dissociation energy of 135 kcal/mol [23]), which easily leads to the target product. Using this approach, target fluorosulfates can be obtained from stable precursors—silyl ethers. However, our comparison, made previously for *para*-substituted 2-phenylbenzoxazoles, showed that the use of O-TBS substituted derivative as a substrate for the SuFEx reaction led to a low yield of **BOSp** (17%), while with the non-silylated **BOp** substrate (Scheme 1), the 59% yield of **BOSp** was achieved [19]. For comparative purposes, we have also synthesized the silyl ether **BOmTBS** in 86% yield (Scheme 2) using *tert*-butyldimethylsilyl chloride (TBSCl) as the silylating agent. It should be noted that our attempts to obtain *ortho*-OTBS derivative of 2-phenylbenzoxazole from **BOo** and TBSCl did not lead to a noticeable conversion of the starting compounds, possibly due to steric hindrances and peculiarities of the OH-group interaction with the nitrogen atom of benzoxazole [24].

**Scheme 3:**
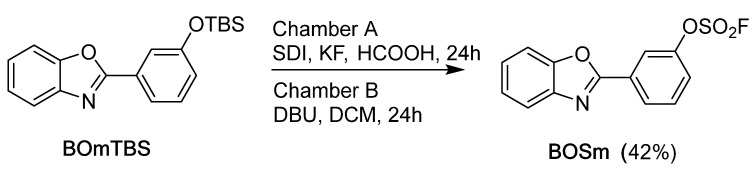
The using of silyl ether derivatives of benzoxazole for synthesis of fluorosulfate derivatives.

Furthermore, we used the silyl ether **BOmTBS** as a substrate for SuFEx reaction (Scheme 3) and synthesized **BOSm** in 42% yield, which was lower than with the non-silylated substrate **BOm** (53%, Table 1).

The experimental data demonstrate that the preliminary silylation of 2-(hydroxyphenyl)benzoxazole substrates is not reasonable, resulting in diminished yields of the desired SuFEx coupling product. This observation can be attributed to the enhanced reactivity of the silylated substrate, which exhibits increased propensity for side reactions with the formed fluorosulfate derivative. Consequently, this elevated reactivity promotes undesired dimerization pathways, leading to the formation of dimeric by-products rather than the intended target molecule.

### 2.2. Single-Crystal X-Ray Analysis

In this paper, we report the first preparation of the *meta*-substituted 2-phenylbenzoxazole fluorosulfate (**BOSm**). In addition to the NMR, IR, and LC/MS data (see Materials and Methods), the newly synthesized derivative was characterized by single-crystal X-ray analysis.

**Figure 2:**
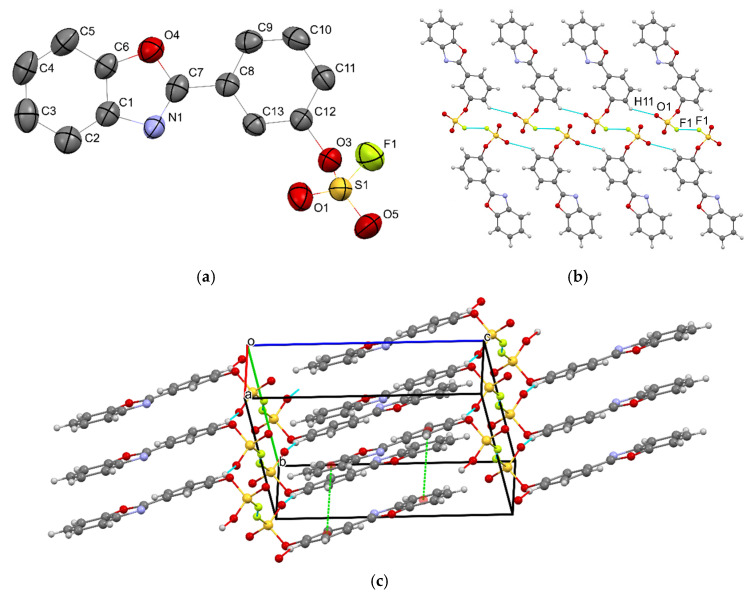
Crystal structure of compound **BOSm**. (**a**) Molecular structure of **BOSm** showing the atom labeling scheme; thermal ellipsoids are drawn at 50% probability level; hydrogen atoms are omitted for clarity. (**b**) Supramolecular chains formed by F···F and C–H···O intermolecular interactions (shown as dashed cyan lines). (**c**) π-stacking interactions between the molecules of **BOSm**.

Compound **BOSm** crystallizes in a triclinic crystal system, space group P–1. The asymmetric unit consists of one formula unit and the unit cell contains two molecules of **BOSm** (Figure 2a). Selected bond lengths are given in Table 2.

The aromatic system of the molecule is nearly planar, with the torsion angle corresponding to the rotation around the C–C bond joining the aromatic cycles of only 3.2°. The molecules of compound **BOSm** form two types of intermolecular interactions, both of which involve the fluorosulfate group (Figure 2b): (i) short F···F contacts (d(F···F) = 2.843 Å); (ii) C–H···O contacts (d(H···O) = 2.658 Å, <(C–H···O) = 130.7°). The F···F internuclear distance is less than the sum of van der Waals radii [25] by 0.097 Å and the angle <(S–F···F) = 153.1° is close enough to 180° to describe this interaction as a halogen bond according to IUPAC criteria [26]. The indicated contacts join the pairs of molecules of compound **BOSm** into supramolecular chains oriented along the crystallographic axis a (Figure 2b). Upon packing of the chains in the crystal structure, 2-phenylbenzoxazole units are oriented in a head-to-tail fashion and participate in π-stacking interactions between the phenyl rings and the phenylene ring of benzoxazole moiety (Figure 2c); the distance between the least-squares planes of the molecules is 3.557 Å and ring centroid to centroid distance is 3.801 Å.

### 2.3. Cytotoxic Activity

Considering that numerous compounds containing the benzoxazole moiety possess anticancer properties (see Introduction), we investigated the cytotoxic activity of **BOo**, **BOm**, and **BOp** and their fluorosulfate derivatives **BOSo**, **BOSm**, and **BOSp**.

Initial screening of antiproliferative activity revealed a moderate dose-dependent inhibitory effect on tumor cell proliferation. Significant antiproliferative activity was observed at high concentrations (50–200 μM), whereas lower concentrations (3.1–25 μM) did not demonstrate statistically significant inhibition of cell proliferation (*p* > 0.05). Notably, the majority of the investigated compounds (**BOm**, **BOp**, **BOSm**, **BOSp**) exhibited predominantly cytostatic effects, characterized by reversible inhibition of cell proliferation without inducing cell death. Despite the similar structure of the compounds, pronounced cytotoxic effects, accompanied by morphological signs of cellular destruction, were observed only for compounds **BOo** and **BOSo**, with dose-dependent decrease in cell monolayer density, and alterations in cellular morphology were observed, correlating with quantitative viability assessments by MTT test.

Comparative analysis of different cell lines revealed heterogeneity in response to the test compounds. Breast cancer cell lines (MCF-7 and BT-474) demonstrated significantly higher susceptibility to the cytostatic actions of all tested substances compared to prostate cancer cells (PC-3), which was relatively resistant (Figure S2). Normal fibroblasts also exhibited enhanced resistance to cytotoxic effects, suggesting a certain type of selective activity of the investigated compounds against some types of tumor cells.

Extended investigation of all compounds against most susceptible breast cancer cell lines with increased exposure time (48 h) and expanded concentration range (up to 400 μM) provided more detailed characterization of antiproliferative effects. Compound **BOSo** demonstrated the most pronounced cytotoxic activity, inducing cell death at maximum concentrations after 48 h incubation. For the remaining compounds, complete suppression of growth and death of the cell population was not achieved, even at the maximum concentration of 400 μM. Further concentration increases were limited by compound solubility under physiological conditions.

Quantitative assessment of cytotoxic activity enabled the calculation of half-maximal inhibitory concentration (IC_50_) values for compound **BOSo** and the extrapolation of predicted values for other compounds using non-linear regression analysis (Table 3).

The obtained results demonstrate predominantly cytostatic effects of the investigated compounds only at maximum studied concentrations, approaching their solubility limit. However, the cytotoxic effect of compound **BOSo** may be of interest for potential therapeutic applications as an antiproliferative agent with relatively low cytotoxicity.

Notably, the absence of significant cytostatic and cytotoxic effects for all the studied compounds at concentrations below 25 μM indicates a clear concentration threshold for their cytostatic activity. This observation has substantial implications for the potential therapeutic window and safety assessment of the investigated compounds.

A remarkable feature of compounds **BOSo-p**, containing the fluorosulfate group, possess inherent fluorescence, which opens additional options for practical applications. Compounds **BOSm** and **BOSp** are of particular interest in this field, demonstrating an optimal combination of low cytotoxicity and stable luminescent characteristics. The identified properties provide a solid base for further structural optimization to enhance target properties while minimizing undesirable effects.

Thus, the investigated compounds demonstrate significant potential for the development of novel multifunctional molecular tools combining biological activity with potent fluorescent visualization capabilities (see below), thereby opening new possibilities in chemical biology, molecular diagnostics, and personalized medicine.

The integration of cytostatic properties with inherent fluorescence characteristics presents unique opportunities for real-time monitoring, potentially advancing our understanding of drug–cell interactions and facilitating the development of more effective targeted therapies. Further studies focusing on structure–activity relationships and molecular mechanisms of action will be essential for optimizing these compounds for specific biomedical applications.

### 2.4. The Docking Study

The human estrogen receptor (hER) is expressed in breast cancer MCF-7 cells and plays a major role in tumorigenic processes [27]. This receptor was previously investigated as a target for compounds with cytotoxic activity against the breast cancer MCF-7 cell line [28]. The human epidermal growth factor receptor 2 (HER2) is a protein that also plays a significant role in the development and progression of breast cancer [29,30]. We have chosen these biotargets for in silico investigation of the benzoxazole derivatives as potential anticancer agents. Additionally, we considered the possibility of the studied benzoxazoles to intercalate with DNA analogously to doxorubicin and other molecules containing planar fragments.

**Figure 3:**
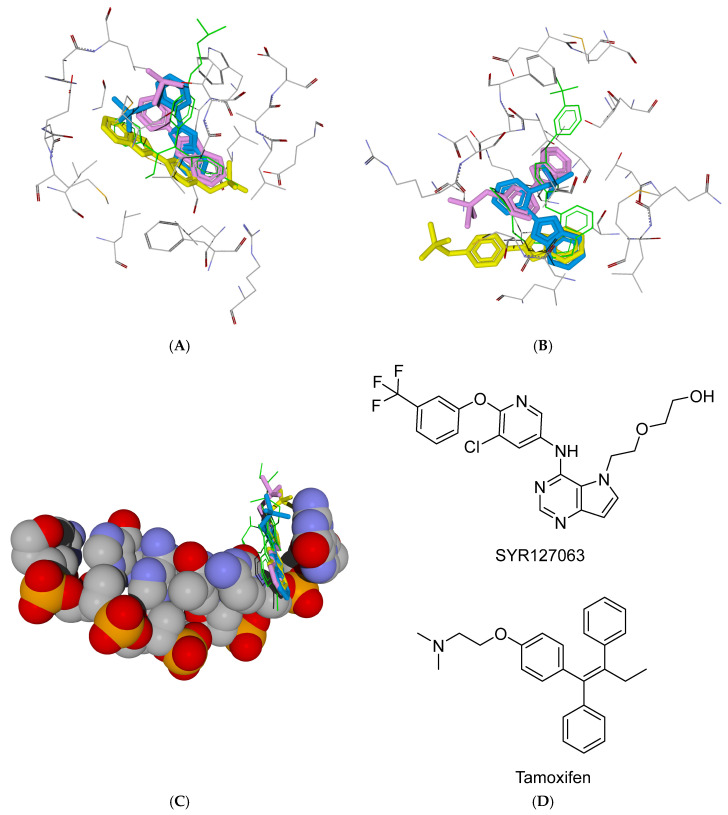
The superimposed docking poses of compounds **BOSo** (blue sticks), **BOSm** (pink sticks), and **BOSp** (yellow sticks) located in the binding sites of hER (PDB: 3ERT, Panel (**A**)) and HER2 (PDB: 3PP0, Panel (**B**)) and intercalated between G and C bases of DNA strand (PDB: 151D, Panel (**C**)). The cocrystallized ligands tamoxifen, SYR127063, and doxorubicin are shown in thin green sticks on panels (**A**), (**B**), and (**C**), respectively. The hER and HER2 residues within 3 Å from the cocrystallized ligands are visible. The structures of tamoxifen and SYR127063 are shown in panel (**D**).

We have performed a docking study for the investigated benzoxazoles into the binding sites of hER (PDB: 3ERT) and HER2 (PDB: 3PP0) and between the nucleotide bases of DNA (PDB: 151D), using the ROSIE server [31]. The obtained docking poses are shown in Figure 3 (superimposed structures of fluorosulfate derivatives) and Figures S3–S5. All the ligands are anchored within the binding sites of hER and HER2 by hydrogen bonding interactions and are characterized by significant Interface Energy scores in the range of −12…−16 kcal/mol (Table 4). However, the binding modes of the fluorosulfate derivatives are noticeably different. Thus, compound **BOSo**, which is cytotoxic on MCF-7 (Table 3), demonstrated spatial similarity to tamoxifen, a known antagonist of the hER receptor, overlapping well with key bulky pharmacophore areas of the antagonist (Figure 3A). Analogously, the docking pose of **BOSo** in the HER2 binding site significantly overlaps with the hydroxyethyl, pyrimidine, and pyridine moieties of the selective HER2 inhibitor SYR127063 cocrystallized in 3PP0 structure (Figure 3B). Low-cytotoxic benzoxazoles **BOSm**, **BOSp**, **BOo**, **BOm**, and **BOp** did not reveal a high match to the positions of the cocrystallized tamoxifen and SYR127063 molecules (Figure 3A,B and Figures S3 and S4). Finally, the docking results demonstrate the possibility of the investigated benzoxazole derivatives to intercalate between neighboring guanine and cytosine bases of DNA (Table 4, Figure 3C and Figure S5), similarly to the well-known doxorubicin intercalator. The fluorosulfate groups of **BOSo**, **BOSm**, and **BOSp** form hydrogen bonds with the guanine fragment, giving more negative Interface Energy scores on docking to the 151D structure than the corresponding phenol precursors (Table 4).

Our docking results using the potential biotargets (hER, HER2, and DNA) are in agreement with the experimentally obtained data on cytotoxic activity against the breast cancer MCF-7 cell line. The introduction of an *ortho*-fluorosulfate group in the 2-phenylbenzoxazole molecule increased the cytotoxic activity in accordance with the resulting binding modes similar to the known tamoxifen and SYR127063 inhibitors of the hER and HER2 receptors.

### 2.5. Luminescent Properties

The photoluminescent properties of 2-(hydroxyphenyl)benzoxazoles and their fluorosulfate derivatives were studied in the solid state and in acetonitrile solution at room temperature. Both in the solid state and in solution, compounds **BOo**, **BOm**, and **BOp** demonstrate a multiband emission in accordance with complex luminescence mechanisms involving the emission from the higher excited states and excited-state intramolecular proton transfer (ESIPT), previously described for 2-(hydroxyphenyl)benzoxazoles [32,33,34] (Table 5).

**Figure 4:**
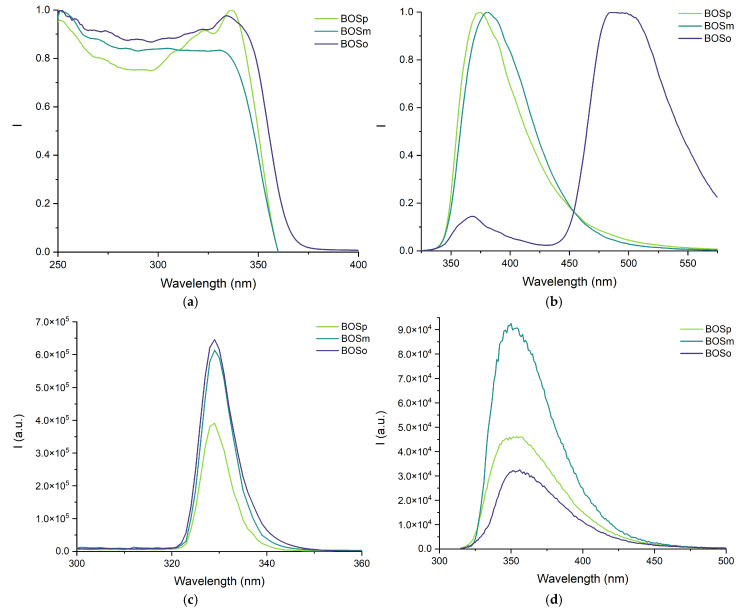
Excitation and emission spectra of 2-(fluorosulfatophenyl)benzoxazoles (**BOSo**, **BOSm,** and **BOSp**): (**a**) normalized solid-state excitation spectra, λ_em_ = 375 nm (**BOSp**), 380 nm (**BOSm**), 485 nm (**BOSo**); (**b**) normalized solid-state emission spectra, λ_ex_ = 300 nm; (**c**) excitation spectra in acetonitrile solution (1.0·10^−3^ M), λ_em_ = 375 nm (**BOSp**), 350 nm (**BOSm**), 360 nm (**BOSo**); (**d**) emission spectra in acetonitrile solution (1.0·10^−3^ M), λ_ex_ = 300 nm.

The photoluminescent properties of the fluorosulfate derivatives differ greatly from their 2-(hydroxyphenyl)benzoxazole counterparts. Thus, only wide bands covering all of the UV region from 250 to 350 nm were observed in their excitation spectra (Figure 4a). Compounds **BOSp** and **BOSm** exhibited emission exclusively in the ultraviolet region. Their emission maxima occurred at 375 nm and 381 nm, respectively. These wavelengths represent a hypsochromic shift relative to compounds **BOp** and **BOm** (Figure 4b). In contrast, compound **BOSo** displayed intense fluorescence in the visible spectrum. Its primary emission maximum was observed at 494 nm. Additionally, this compound showed a minor emission band in the UV region at approximately 368 nm (Figure 4b).

It is interesting to note that in acetonitrile solution, the photoluminescent properties of all three fluorosulfate derivatives are almost identical—they feature a single excitation band at 329 nm (Figure 4c) and a single narrow emission band near 350 nm (Figure 4d). The excited-state lifetimes were in a low nanosecond range characteristic of fluorescence (Figures S6 and S7, Table 5). The photoluminescence quantum yields were high enough both in the solid state and in solution, with the highest values found for 4-fluorosulfate derivative **BOSp**, and being up to 64% in acetonitrile (Table 5).

Since the luminescent properties under conditions mimicking physiological ones are important for biorelevant applications, the excitation and emission spectra of 2-(hydroxyphenyl)benzoxazoles and their fluorosulfate derivatives were additionally studied in phosphate buffer solution (PBS). All six compounds retained their luminescent properties upon their introduction to PBS. Similarly to acetonitrile solution, 2-(o-hydroxyphenyl)benzoxazole (**BOo**) in PBS demonstrated a multiband emission profile with a slight bathochromic shift in the emission maxima (Table 5, Figure S8). *Para*- and *meta*-derivatives **BOp** and **BOm** in PBS revealed single-band emission profiles with the maxima at 359 and 363 nm, correspondingly. The emission spectrum of 2-(2-(fluorosulfato)phenyl)benzoxazole (**BOSo**) in PBS demonstrates a strong emission band near 495 nm (Table 5, Figure S8), which was not observed in acetonitrile solution, while the spectra of **BOSp** and **BOSm** compounds in PBS are similar to those in acetonitrile, only with a bathochromic shift in the emission maxima (Table 5, Figure S8). The emission intensity of **BOo** and **BOm** in PBS was insufficient to accurately measure the quantum yields and the excited-state lifetimes. For the rest of the studied derivatives, the photoluminescence quantum yields in PBS were very close to those measured in acetonitrile (Table 5); likewise, the excited-state lifetimes in PBS were in the low nanosecond range characteristic of S_1_→S_0_ fluorescence (Table 5, Figure S9).

### 2.6. DFT Calculations of the Luminescent Properties

In order to gain insight into the nature of electronic transitions associated with the absorption and emission processes, calculations of the luminescence characteristics of fluorosulfate benzoxazole derivatives were performed. The calculations employed time-dependent density functional theory (TDDFT) using Gaussian 16 software. Ground state molecular geometries were optimized via DFT methods. We used the M06-2X hybrid functional with the aug-cc-pVDZ basis set. Solvation effects were modeled using IEFPCM with acetonitrile as the solvent.

**Figure 5:**
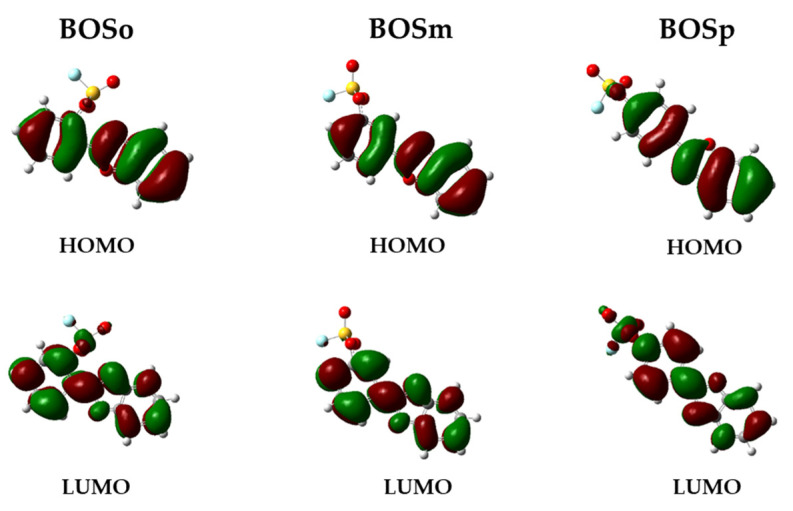
Frontier molecular orbitals of **BOS** derivatives.

**Figure 6:**
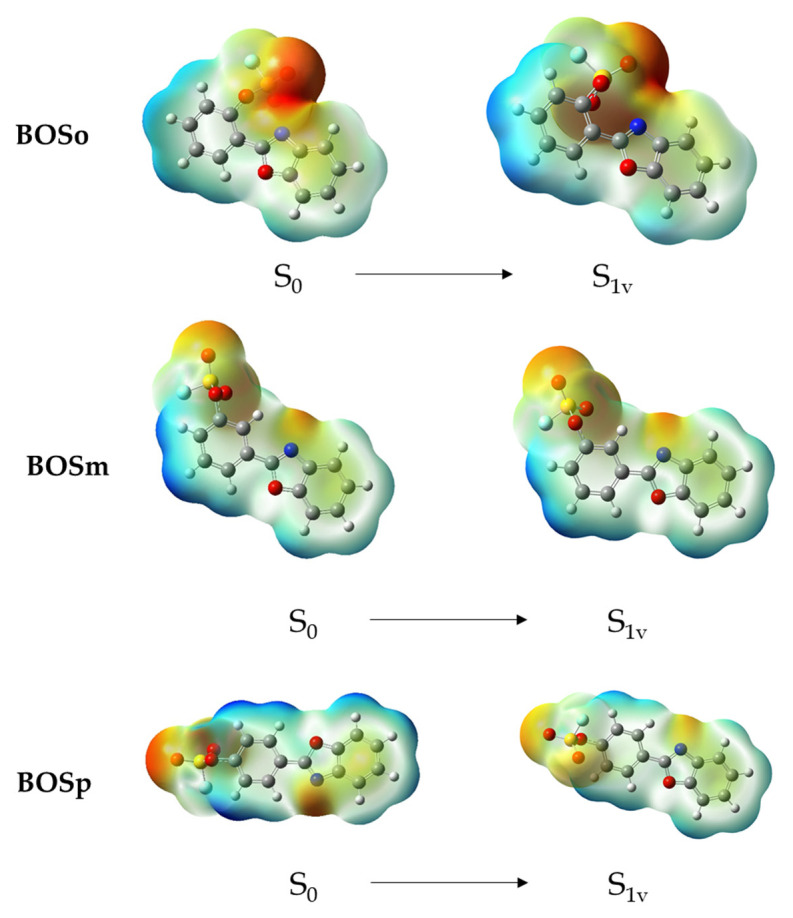
Distributions of electrostatic potential in the ground state S_0_ and the vertical excited-state S_1v_.

Both the HOMO and LUMO of the fluorosulfate derivatives in the ground and excited states show distinct π-character (Figure 5), indicating that the S_0_→S_1v_ and S_1r_→S_0v_ transitions of **BOSo**, **BOSm**, and **BOSp** possess π,π* characteristics. It can be noted that in all three benzoxazole fluorosulfates, upon transition to an excited state, the electron density shifts toward the substituted phenyl ring. However, the distributions of electrostatic potential in the ground state S_0_ and the vertical excited-state S_1v_ (Figure 6) indicated minor changes on the excitation of the fluorosulfate molecules in acetonitrile solution. The charge separation in **BOSo** is much more pronounced compared to *ortho*- and *meta*-derivatives both in the ground and the excited states (Figure 6), providing an explanation for the considerably higher photoluminescence intensity and the shift in the emission maximum to visible range.

We have found that energies of the vertical transitions S_1r_ → S_0v_ from the relaxed excited state to the non-relaxed ground state (calculated also with the non-equilibrium IEFPCM solvation) present good estimations for the experimental emission maxima in acetonitrile (Table 6).

Unfortunately, the applied TD-DFT approximation is incapable of reproducing the fluorescence results obtained experimentally for the solid-state samples, while these results are of great interest. For example, the unique **BOSo** fluorescent properties in the solid phase, contrasting with the *meta*- and *para*-isomers (Figure 4b), give promising opportunities for the development of selective fluorescent probes. The chemical reactivity of the OSO_2_F group and its potential for covalent surface modification and functionalization of macromolecules further enhance the practical applications of these benzoxazole derivatives.

## 3. Materials and Methods

### 3.1. General Information and Synthesis of Compounds

The reagents used in the experiment were obtained from Sigma-Aldrich (Burlington, MA, USA) and Acros Organics (Geel, Antwerpen, Belgium). LC/MS analyses were performed using an Agilent Infinity liquid chromatograph (Santa Clara, CA, USA) equipped with an Accurate Mass QTOF 6530 mass spectrometer (Santa Clara, CA, USA). Chromatographic conditions: column Zorbax EclipsePlusC18 (Agilent Technologies, Santa Clara, CA, USA) 1.8 μm, 2.1 × 50 mm; eluent H_2_O—acetonitrile (15:85%, *v*/*v*); flow rate 0.2 mL/min. Ionization source: ESI in positive mode. The NMR ^1^H, ^13^C, and ^19^F spectra were acquired using a Bruker AVANCE III HD spectrometer (Bruker corporation, Billerica, CA, USA) operating at 400 MHz for ^1^H, 100 MHz for ^13^C, and 376 MHz for ^19^F. Melting points of the synthesized compounds were determined with an SMP30 Melting Point Apparatus (Buch Holm, Herlev, Denmark) at a heating rate of 2.5 °C per minute. Thin-layer chromatography (TLC), employing Merck silica gel 60 F_254_ plates (Merck KGaA, Darmstadt, Germany), was used to monitor the progress of reactions. Purification via column chromatography was conducted using Silica Gel 60 (Merck KGaA, Darmstadt, Germany), with a particle size range of 0.040–0.063 mm.

The NMR spectra of the 2-phenylbenzoxazole derivatives are shown in Figures S10–S20. The atom numbering schemes for molecules **BOmTBS** and **BOSm**, employed in the assignment of their ^1^H NMR signals, are depicted in Figures S10 and S12. The mass spectra of the fluorosulfate derivatives are presented in Figures S21–S23.

The compounds **BOo**, **BOm**, and **BOp** were synthesized using methodologies outlined in prior studies [35]. The synthesis of compound **BOpTBS** was performed using the experimental procedure from paper [19].

*2-(3-(tert-Butyldimethylsilyloxy)phenyl)benzoxazole* (**BOmTBS**).

To a vigorously stirred mixture of **BOm** (106 mg, 0.5 mmol) and imidazole (68 mg, 1 mmol) dissolved in dichloromethane (DCM, 2 mL), a solution of *tert*-butyldimethylsilyl chloride (TBSCl, 113 mg, 0.75 mmol) in DCM (1 mL) was slowly added dropwise at 0 °C. The reaction was subsequently allowed to reach ambient temperature and stirred for 3 h, with progress monitored via TLC (hexane–ethyl acetate, 8:2). Upon completion, the mixture was quenched by pouring into a saturated aqueous sodium bicarbonate solution and extracted with DCM. The organic extract was dried using anhydrous sodium sulfate, filtered, and concentrated under reduced pressure. Brown oil. Yield 86%. ^1^H NMR (DMSO-d_6_), δ, ppm: 7.80 (m, 3H, H-1, H-4, H-8), 7.61 (s, 1H, H-5), 7.50 (t, *J* = 8 Hz, 1H, H-7), 7.45-7.39 (m, 2H, H-2, H-3), 7.12 (dd, *J* = 8.3, 2.4 Hz, 1H, H-6), 0.98 (s, 9H, *t*-Bu), 0.24 (s, 6H, Si(CH_3_)_2_). ^13^C NMR (DMSO-d_6_), δ, ppm: 161.9, 155.6, 150.2, 141.4, 130.8, 127.8, 125.6, 124.9, 123.5, 120.5, 119.9, 118.2, 111.0, 25.5, 17.9, −4.6. Found, %: C 70.24, H 7.05, N 4.41. C_19_H_23_NO_2_Si. Calculated, %: C 70.11, H 7.12, N 4.30. GC-MS *m*/*z* 325.2 (27.2%, M^+●^).

Compounds **BOSo** [18] and **BOSp** [19] were prepared according to the methods described previously.

*2-(3-(Fluorosulfato)phenyl)benzoxazole* (**BOSm**).

In a small two-chamber reactor (Figure S1), chamber A was charged with 1,1′-sulfonyldiimidazole (SDI, 495 mg, 2.5 mmol) and potassium fluoride (378 mg, 6.5 mmol). Chamber B was filled with compound **BOm** (106 mg, 0.5 mmol), 1,8-diazabicyclo[5.4.0]undec-7-ene (DBU, 0.3 mL, 2.0 mmol), and dichloromethane (DCM, 3 mL). Both chambers were sealed tightly, and either formic acid or trifluoroacetic acid (1.6 mL) was injected through the septum in chamber A to generate SO_2_F_2_ gas. Following 24 h of stirring at ambient temperature, the reaction mixture from chamber B was transferred to a 100 mL round-bottom flask. The chamber was rinsed with an additional 4 mL of DCM, and the combined organic solution was concentrated under reduced pressure. The crude residue was purified by column chromatography on silica gel using a hexane–ethyl acetate (1:1) eluent to isolate the target product. Colorless crystals. Yield 53% (with the use of formic acid) or 24% (with the use of TFA). The yield of compound **BOSm** was 42% when **BOmTBS** was used as a substrate. M.p. 106.5–108 °C. ^1^H NMR (CDCl_3_), δ, ppm: 8.30 (d, *J* = 7.6 Hz, 1H, H-8), 8.22 (s, 1H, H-5), 7.80–7.77 (m, 1H, H-1), 7.66–7.59 (m, 2H, H-4, H-7), 7.49 (dd, *J* = 7.6, 1.6 Hz, 1H, H-6), 7.42–7.36 (m, 2H, H-2, H-3). ^13^C NMR (CDCl_3_), δ, ppm: 160.7, 150.8, 150.3, 141.8, 131.2, 129.8, 127.6, 126.0, 125.1, 123.7, 120.4, 120.1, 110.9. ^19^F NMR (CDCl_3_), δ, ppm: 38.4. LC/MS (ESI^+^); m/z: 294.0231 [M + H]^+^ experimental ([C_13_H_8_FNO_4_S + H]^+^ = 294.0236 theor.).

### 3.2. X-Ray Crystal Structure Determination

Single-crystal X-ray diffraction data for compound **BOSm** were acquired at 290(2) K using an automated Agilent Xcalibur four-circle diffractometer (Santa Clara, CA, USA) equipped with an AtlasS2 area detector (Agilent Technologies, Santa Clara, CA, USA). The experiment employed MoKα radiation (λ = 0.71073 Å), monochromated via a graphite crystal. Absorption corrections were applied using the SADABS 2.11 software package [36]. The crystal structures were solved and refined with the SHELXT-2019 [37] and SHELXL-2019 [38] programs. Anisotropic thermal displacement parameters were refined for all non-hydrogen atoms, while hydrogen atoms were positioned geometrically and refined using the riding model. Key crystallographic parameters and refinement statistics are shown in Table 7.

### 3.3. Assessment of Cell Viability and Cytotoxicity

#### 3.3.1. Cell Culture and Maintenance

Human cancer cell lines were procured from PrimeBioMed LLC (Moscow, Russia). These included PC-3 cells (human prostate adenocarcinoma-derived), BT-474 cells (human breast carcinoma-derived), and MCF-7 cells (human breast adenocarcinoma-derived). Additionally, murine 3T3-L1 embryonic fibroblasts were obtained from the same supplier. All cell lines were cultured and maintained in Dulbecco’s Modified Eagle Medium (DMEM), supplemented with 10% FBS and antibiotics (all from PanEco Ltd., Moscow, Russia). Cells were cultured under standardized conditions at 37 °C in a humidified atmosphere containing 5% CO_2_ using a CB-170 incubator (Binder GmbH, Tuttlingen, Germany). All experiments were conducted using cells in their exponential growth phase.

#### 3.3.2. Cell Treatment

All the cell cultures were seeded at a density of 5 × 10^3^ cells per well in 96-well microplates (SPL Life Sciences Co Ltd, Pocheon-si, South Korea). Cell concentrations were determined using an automated cell counter. Cells were allowed to adhere for 24 h under standard incubation conditions. These conditions included a temperature of 37 °C, 5% CO_2_ atmosphere, and saturated humidity. After the attachment period, the culture medium was aspirated and replaced with fresh medium. The fresh medium contained serial dilutions of the test compounds at predetermined concentrations. For general cytotoxicity screening, compounds were evaluated at concentrations ranging from 3.1 to 200 μM (3.1, 6.25, 12.5, 25, 50, 100, and 200 μM), with a 24 h exposure period. In the final stage of the experiment, an extended range of concentrations up to 400 μM (6.25, 12.5, 25, 50, 100, 200, and 400 μM) with an extended exposure period of 48 h was used to assess potential time- and dose-dependent effects for most sensitive lines (BT-474 and MCF-7).

#### 3.3.3. MTT Viability Assay

Cell viability was assessed using the MTT (3-(4,5-dimethylthiazol-2-yl)-2,5-diphenyl-2*H*-tetrazolium bromide) colorimetric assay. Following the treatment period, the culture medium was carefully aspirated and replaced with 100 μL of MTT reagent. The cells were subsequently incubated for 4 h at 37 °C under 5% CO_2_ atmosphere. After incubation period, the resulting formazan crystals were dissolved by adding 100 μL of dimethyl sulfoxide. Optical density measurements were performed at 570 nm wavelength using a microplate reader. The absorbance values were recorded and used for subsequent viability calculations. The parameters of the control group of cells (without exposure to compounds) were taken as 100% viability.

#### 3.3.4. Statistical Data Processing

The results were expressed as mean ± standard deviation (SD). Statistical analysis was performed using GraphPad Prism software (version 9.0, GraphPad Software Inc., San Diego, CA, USA). Half-maximal inhibitory concentration (IC_50_) values were calculated using the non-linear regression analysis function in GraphPad Prism.

### 3.4. Molecular Docking

The 3D molecular models of ligands **BOo**, **BOm**, **BOp**, **BOSo**, **BOSm**, and **BOSp** were generated using ChemOffice 2016 software. These structures were pre-optimized with the MM2 force field and exported in the SDF format. The hER, HER2, and DNA structures were downloaded from PDB (entries 3ERT [28], 3PP0 [39], and 151D [40], respectively). These structures along with prepared molecular models of the investigated benzoxazoles were uploaded on the ROSIE web server [31], where the ligand docking protocol [41,42] was applied. The search areas for docking were positioned at the geometric centroid of the co-crystallized tamoxifen (3ERT), SYR127063 (3PP0), or doxorubicin (151D) ligands in the PDB structures. Up to 500 conformers were generated for each ligand with the use of the BCL conformer generation algorithm [43] incorporated in the ROSIE ligand docking protocol. Each compound underwent 2000 docking runs. The lowest-energy docking poses were retained and evaluated using the Molegro Virtual Docker 6.0 (MVD) software (CLC Bio, Copenhagen, Denmark).

### 3.5. Luminescence Measurements

Luminescence spectra were recorded using HORIBA Fluorolog 3 spectrofluorimeter. The instrument was equipped with a 450 W ozone-free xenon arc lamp serving as the excitation source. Emitted photons were detected using a PC177CE-010 detection module fitted with an R2658 photomultiplier tube. Absolute quantum yields were determined using a Quanta-ϕ integrating sphere (HORIBA Jobin Yvon SAS, Edison, NJ, USA). Excitation and emission spectra were corrected for source intensity (lamp and diffraction grating) and emission spectral response (detector and diffraction grating) by standard correction curves. The emission decay kinetics were monitored using the time-correlated single-photon counting (TCSPC) technique and a set of solid-state lasers of various wavelengths. The powdered samples were placed between two non-fluorescent quartz plates and the spectra of the compounds in acetonitrile and in phosphate buffer solution (pH 7.4) were recorded in 1 cm quartz cuvettes.

### 3.6. DFT and TDDFT Calculations

The optimized ground-state molecular structures of the studied compounds were determined using density functional theory (DFT) calculations with the M06-2X exchange-correlation functional [44] and the aug-cc-pVDZ basis set [45]. Solvent effects of acetonitrile were modeled using the IEFPCM approach [46]. Excitation energies and oscillator strengths for the first six singlet excited states were calculated at the time-dependent DFT (TDDFT) level, using the same functional and basis set as in the ground-state optimization. Subsequently, the geometry of the first singlet excited state was optimized in acetonitrile using the IEFPCM solvation model. The nature of the stationary points (energy minima) for both ground and excited states was verified through vibrational frequency analysis. Fluorescence emission energy (de-excitation energy) was computed as the energy difference between the relaxed S_1_ excited state and the non-relaxed S_0v_ ground state (with non-equilibrium solvation applied to S_0v_). All computations were carried out using the GAUSSIAN 16 software suite [47].

## 4. Conclusions

Summarizing the results, the preparation of 2-(3-(fluorosulfato)phenyl)benzoxazole was reported for the first time. Novel features of the synthesis were studied, namely, the method of SO_2_F_2_ generation and the influence of the OH group pre-silylation. A series of 2-(hydroxyphenyl)benzoxazoles and 2-(fluorosulfatophenyl)benzoxazoles were evaluated for their antiproliferative effects on tumor cells in vitro, with notable growth inhibition of PC-3 prostate adenocarcinoma cells as well as BT-474 and MCF-7 breast carcinoma cell lines. MCF-7 breast cancer cells exhibited particularly high sensitivity to the cytostatic actions of all the tested benzoxazole compounds compared to the other cell lines. It has been shown that the introduction of a fluorosulfate group into *ortho*-position (compound **BOSo**) results in potent cytotoxic activity. This activity is in accordance with the docking results showing the spatial similarity of **BOSo** to tamoxifen and SYR127063, an established hER and HER2 antagonist, respectively. Compound **BOSo** demonstrates significant potential as an antiproliferative therapeutic agent. The photoluminescent properties of the fluorosulfate derivatives were investigated in the solid state and in acetonitrile solution. The highest value of photoluminescence quantum yield (up to 64% in acetonitrile) was found for *para*-fluorosulfate derivative **BOSp**. In the solid state, compound **BOSo** has a strong luminescence in the visible region, in contrast to the corresponding *meta*- and *para*-isomers. Thus, the investigated compounds demonstrate significant potential for the development of novel multifunctional molecular tools combining biological activity with fluorescent visualization capabilities.

## Figures and Tables

**Table 1:** Yields of compounds **BOSm** and **BOSp** (Scheme 1) with the use of trifluoroacetic acid (TFA) or formic acid to produce SO_2_F_2_.

Acid	2-(3-(fluorosulfato)phenyl)benzoxazole	2-(4-(fluorosulfato)phenyl)benzoxazole
TFA	24%	19%
Formic acid	53%	59% [19]

**Table 2:** Selected bond lengths in the structure of **BOSm**.

Bond	Length, Å	Bond	Length, Å
S1–F1	1.5098(15)	C7–C8	1.463(3)
S1–O1	1.4039(18)	O4–C6	1.382(3)
S1–O3	1.5389(17)	O4–C7	1.347(3)
S1–O5	1.4001(18)	N1–C1	1.401(3)
O3–C12	1.442(2)	N1–C7	1.301(3)

**Table 3:** Calculated half-maximal inhibitory concentrations (IC_50_) of different compounds for MCF-7 and BT-474 cells; results presented as mean (95% CI) (μM).

Compounds	BOo	BOm	BOp	BOSo	BOSm	BOSp	Cisplatin
Cell Line							
BT-474	209.3 (190.1–231.7)	256.8 (229.1–291.3)	284.2 (245.6–336.4)	123.3 (119.9–126.8)	426.8 (363.8–527.5)	542.5 (414.5–840.9)	38.5 (36.4–40.7)
MCF-7	122.9 (113.0–133.8)	186.9 (166.9–210.7)	187.1 (169.5–207.6)	87.18 (83.0–91.5)	263.9 (225.4–317.6)	343.1 (302.8–398.6)	33.9 (31.9–36.1)

**Table 4:** The interactions and Interface Energy scores of the investigated benzoxazole derivatives docked into the binding sites of hER (PDB: 3ERT) and HER2 (PDB: 3PP0) and intercalated with DNA (PDB: 151D).

Ligand	Residues (Ligand Groups) Participating in Hydrogen Bonding Interactions and π,π-Stacking with DNA Bases	Interface Energy Score
hER receptor		
**BOo**	Glu353 (OH)	−12.802
**BOm**	Arg394 (OH), Glu353 (OH, weak)	−13.743
**BOp**	Arg394 (OH), Glu353 (OH, weak)	−14.975
**BOSo**	His524 (SO_2_F oxygen atom)	−11.828
**BOSm**	Thr347 (SO_2_F oxygen atom)	−12.135
**BOSp**	Arg394 (SO_2_F oxygen atom)	−11.979
HER2 receptor		
**BOo**	Ser783 (OH, N_Het_), Asp863 (O_Het_, weak)	−16.151
**BOm**	Arg849 (OH), Asn850 (OH), Asp863 (OH, weak)	−14.537
**BOp**	Ser728 (OH), Arg849 (OH), Thr862 (N_Het_)	−16.384
**BOSo**	Thr798 (SO_2_F oxygen atom), Thr862 (PhOSO_2_F oxygen atom)	−12.909
**BOSm**	Arg849 (SO_2_F oxygen atom), Asn850 (SO_2_F oxygen atom, weak; PhOSO_2_F oxygen atom)	−13.853
**BOSp**	Arg849 (SO_2_F oxygen atom), Ser728 (PhOSO_2_F oxygen atom)	−14.863
DNA		
**BOo**	π,π-stacking with G and C bases	−9.505
**BOm**	π,π-stacking with G and C bases	−9.532
**BOp**	π,π-stacking with G and C bases	−9.514
**BOSo**	Guanine (SO_2_F oxygen atom), π,π-stacking with G and C bases	−10.237
**BOSm**	Guanine (SO_2_F oxygen atom), π,π-stacking with G and C bases	−10.202
**BOSp**	Guanine (SO_2_F oxygen atom), π,π-stacking with G and C bases	−9.973

**Table 5:** Photophysical characteristics of 2-(hydroxyphenyl)benzoxazoles and their fluorosulfate derivatives.

Compound	Formula	State	Excitation λ_max_, nm	Emission λ_max_, nm	Θ, %	τ, ns
**BOo**	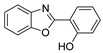	solid	br. 250–350 nm; 428 sh.; 457; 489; 526	480; 503; 537; 571	5	2.2
		MeCN	353 sh.; 435 sh.; 464; 497	427; 517; 544	45	3.4
		PBS	br. 250–350 nm	401; 482; 535; 573	<1%	n.a.
**BOm**	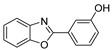	solid	366; 378; 455; 486; 522	410; 430 sh.; 525; 565	<1%	–
		MeCN	334 sh.; 436 sh.; 464; 497	427; 522; 545	28	3.5
		PBS	293	359	<1%	n.a.
**BOp**	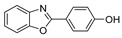	solid	340; 357; 462; 487 sh.; 494; 526	420; 440	<1%	–
		MeCN	334 sh.; 437 sh.; 464; 497	431; 514; 543	23	3.4
		PBS	300	363	24	1.4
**BOSo**	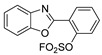	solid	broad 250–350 nm; 334	368, 494	16	6.6
		MeCN	329	354	40	1.4
		PBS	331	363; 495	32	6.2
**BOSm**	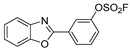	solid	broad 250–350 nm; 334	381	15	1.9
		MeCN	329	349	27	2.0
		PBS	295	361	27	1.7
**BOSp**	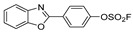	solid	broad 250–350 nm; 337	375	33	8.2 (7%); 5.3 (93%)
		MeCN	329	353	64	1.9
		PBS	298	358	24	1.6

**Table 6:** The calculated and experimental emission wavelengths for the investigated fluorosulfate derivatives of benzoxazole in acetonitrile solution.

Compound	Calculated λ_max_ (nm)	Experimental λ_max_ (nm)	Transition
**BOSo**	343	354	LUMO-HOMO
**BOSm**	346	349	LUMO-HOMO
**BOSp**	350	353	LUMO-HOMO

**Table 7:** Crystallographic data of compound **BOSm**.

Parameter	Value
Empirical formula	C_13_NO_4_SFH_8_
Formula weight	293.26
Temperature, K	290(2)
Crystal system	triclinic
Space group	P-1
a, Å	6.7044(8)
b, Å	7.2299(13)
c, Å	13.1344(17)
α, °	82.644(12)
β, °	79.806(10)
γ, °	86.937(12)
Volume, Å^3^	621.14(16)
Z	2
ρ_calc_, g/cm^3^	1.568
μ, mm^−1^	0.286
F(000)	300.0
Crystal size, mm^3^	0.3 × 0.25 × 0.2
2Θ range for data collection, °	5.684 to 58.068
Index ranges	−9 ≤ h ≤ 8, −7 ≤ k ≤ 9, −17 ≤ l ≤ 17
Reflections collected	5147
Independent reflections	2747 [R_int_ = 0.0192, R_sigma_ = 0.0335]
Data/Restraints/Parameters	2747/0/181
Goodness-of-fit on F^2^	1.052
Final R indexes [I ≥ 2σ (I)]	R_1_ = 0.0484, wR_2_ = 0.1257
Final R indexes [all data]	R_1_ = 0.0650, wR_2_ = 0.1357
Largest diff. peak/hole, e·Å^−3^	0.24/−0.34

## Data Availability

Experimental data associated with this research are available from the authors. Crystallographic data for compound **BOSm** was deposited at the Cambridge Crystallographic Data Centre, CCDC No. 2400927. Copies of the data can be obtained free of charge from the Cambridge Crystallographic Data Centre, 12 Union Road, Cambridge CB2 1EZ, UK (fax: +44-1223-336-033; e-mail: deposit@ccdc.cam.ac.uk).

## Supplementary Materials

The following supporting information can be downloaded at: https://www.mdpi.com/article/10.3390/ijms26157261/s1.
