# Supplementary material for: Luminescent Properties and Cytotoxic Activity of 2-phenylbenzoxazole Fluorosulfate Derivatives
Source: Int J Mol Sci. 2025 Jul 27;26(15):7261. doi: 10.3390/ijms26157261 (PMC12347133; doi:10.3390/ijms26157261)
Supplement: Supplementary file 1 [file ijms-26-07261-s001.zip › ijms-3745934-supplementary.pdf]

Luminescent Properties and Cytotoxic Activity of 2-phenylbenzoxazole Fluorosulfate Derivatives

Nadezhda V. Danilenko [1], Mariia O. Lutsuk [1], Alexey A. Ryadun [2], Dmitry I. Pavlov [2], Evgenii V. Plotnikov [3,4,*], Daria D. Eskova [3], Yulia D. Klimenko [3], Andrei S. Potapov [2] and Andrei I. Khlebnikov [1]

[1] Kizhner Research Center, Tomsk Polytechnic University, 634050 Tomsk, Russia; nadezhda.dani@gmail.com (N.V.D); lutsukma@gmail.com (M.O.L.); aikhl@chem.org.ru (A.I.K.)

[2] Nikolaev Institute of Inorganic Chemistry, Siberian Branch of the Russian Academy of Sciences, 3 Lavrentiev Ave., 630090 Novosibirsk, Russia; pavlov@niic.nsc.ru (D.I.P.), ryadunalexey@mail.ru (A.A.R.), potapov@niic.nsc.ru (A.S.P.)

[3] Research School of Chemistry and Applied Biomedical Sciences, Tomsk Polytechnic University, 30 Lenin Avenue, Tomsk, 634034, Russia; dde5@tpu.ru (D.D.E.), yuliaklim1207@mail.ru (Y.D.K.)

[4] Department of Chemistry, Siberian State Medical University, 2 Moscow Trakt, Tomsk, 634050, Russia

* Correspondence: plotnikov.e@mail.ru (E.V.P.)

Supplementary information

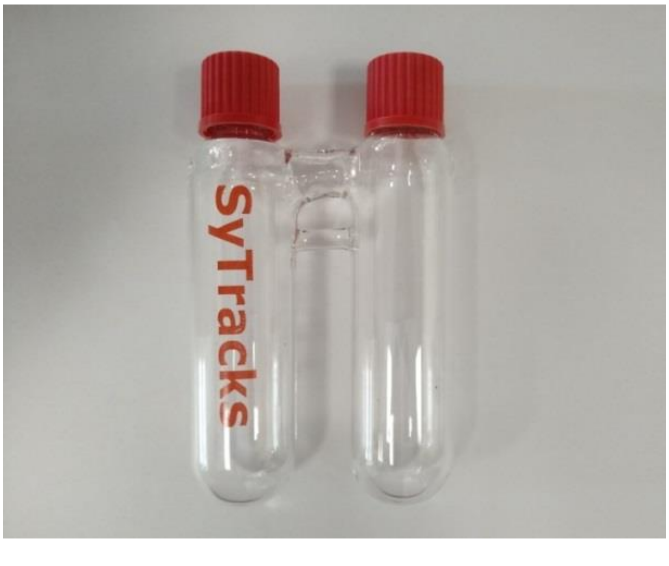

Figure S1. The two-chamber reactor.

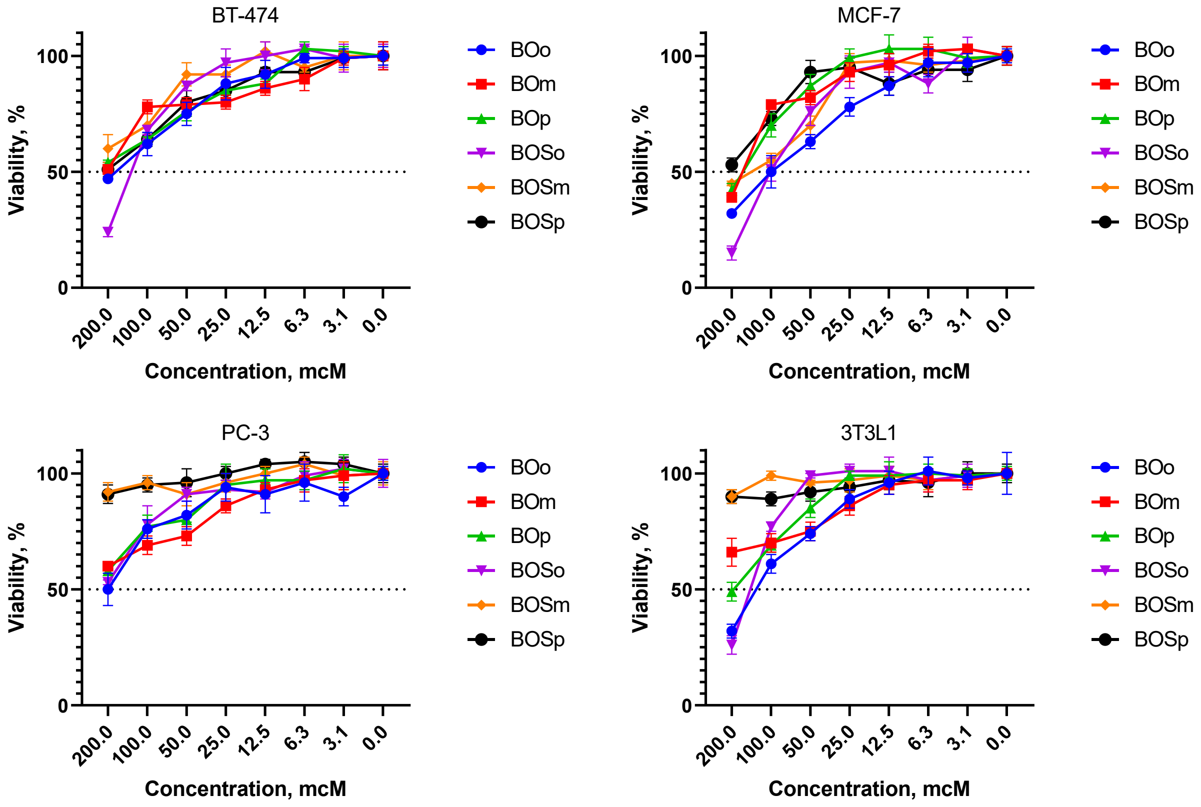

Figure S2. Effect of the tested compounds on the viability of breast cancer, prostate cancer and normal fibroblast cell lines in the concentration range of 0-200 μM.

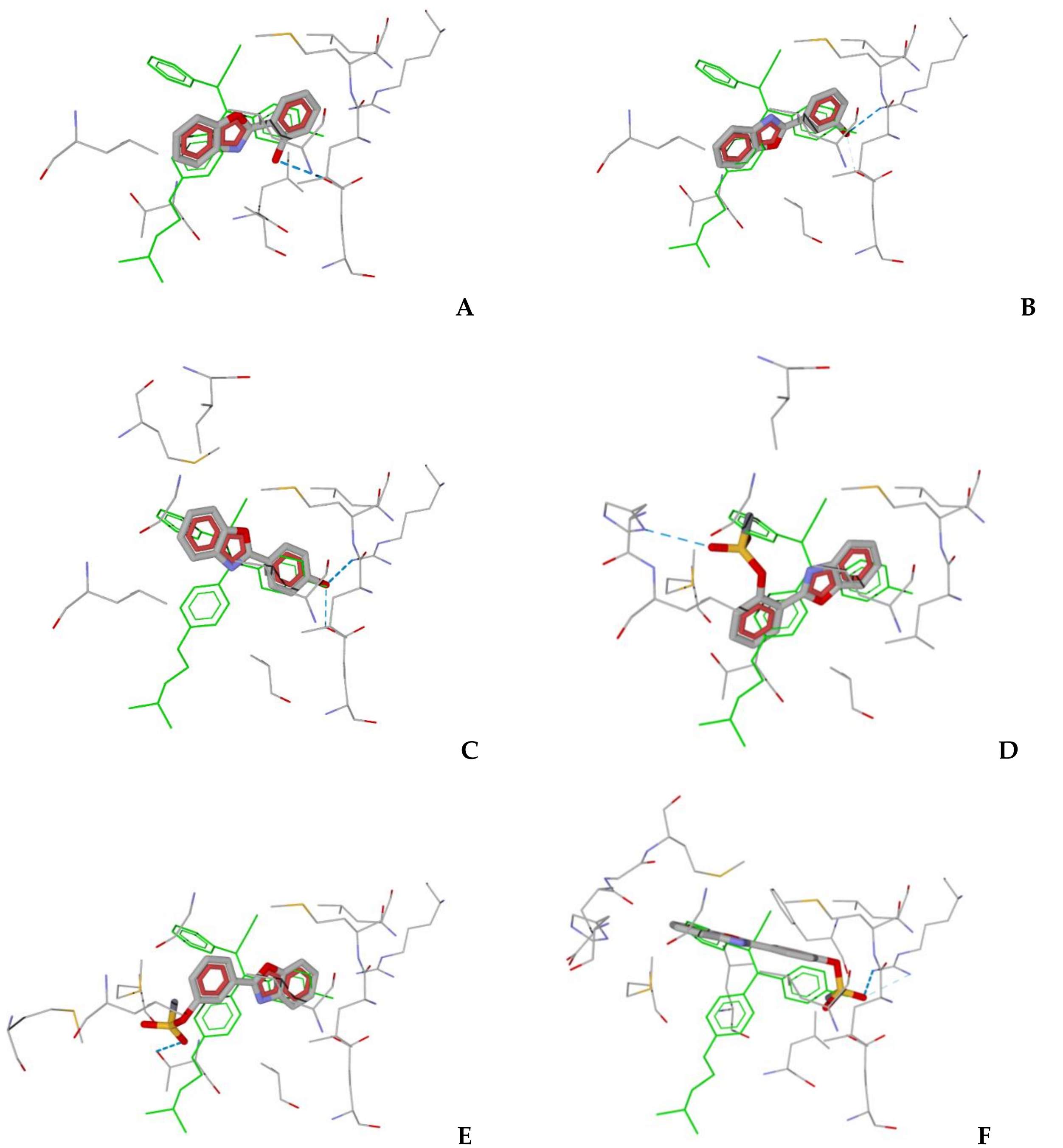

A B C D E F

Figure S3. The docking poses (sticks) of compounds **BOo** (Panel A), **BOm** (Panel B), **BOp** (Panel C), **BOSo** (Panel D), **BOSm** (Panel E), and **BOSp** (Panel F) superimposed on the co-crystallized tamoxifen molecule (thin green sticks) in hER protein (PDB: 3ERT). Hydrogen bonds are shown in dashed blue lines. The residues within 3 Å from each pose are visible.

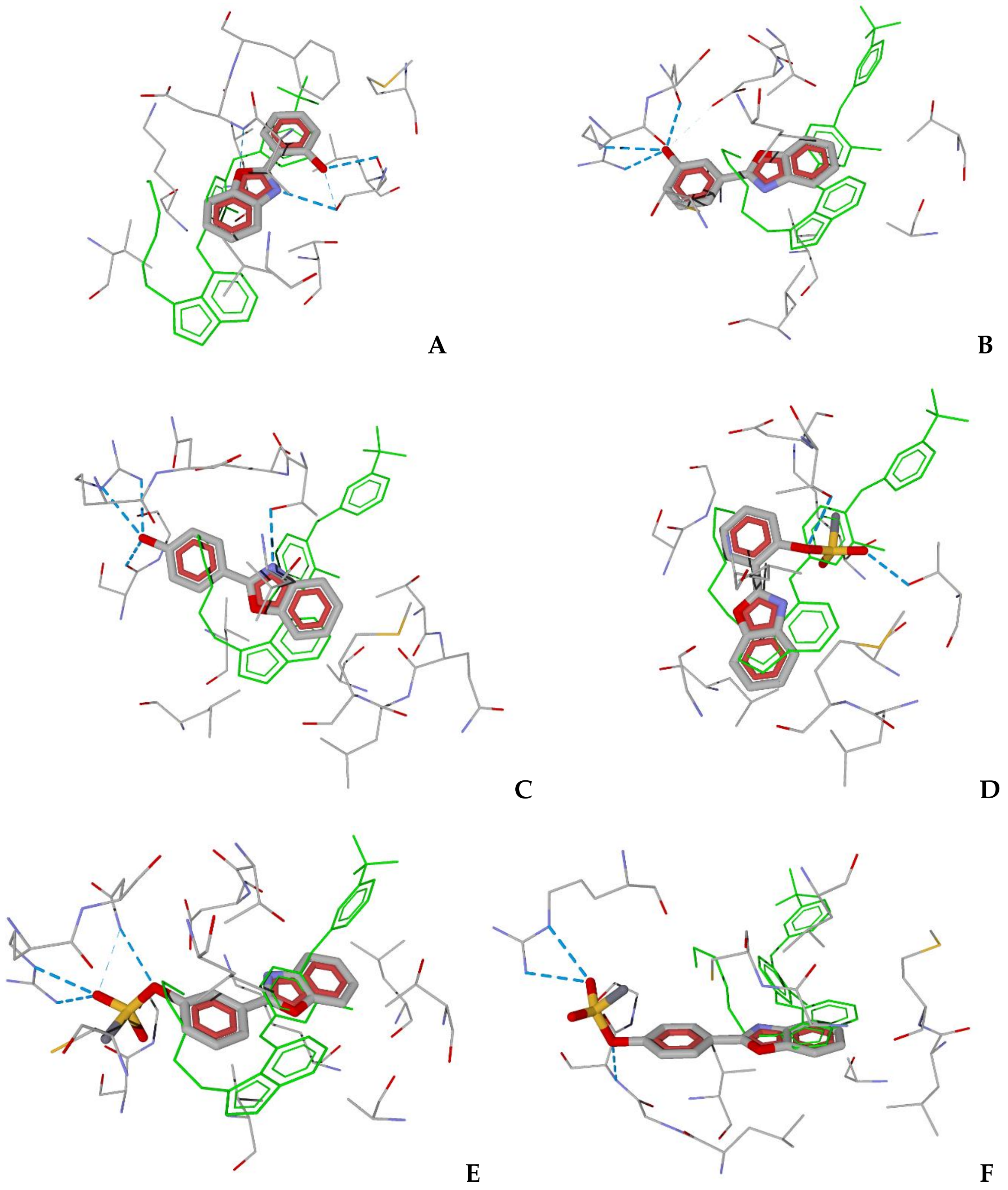

Figure S4. The docking poses (sticks) of compounds **BOo** (Panel A), **BOm** (Panel B), **BOp** (Panel C), **BOSo** (Panel D), **BOSm** (Panel E), and **BOSp** (Panel F) superimposed on the co-crystallized SYR127063 molecule (thin green sticks) in HER2 protein (PDB: 3PP0). Hydrogen bonds are shown in dashed blue lines. The residues within 3 Å from each pose are visible.

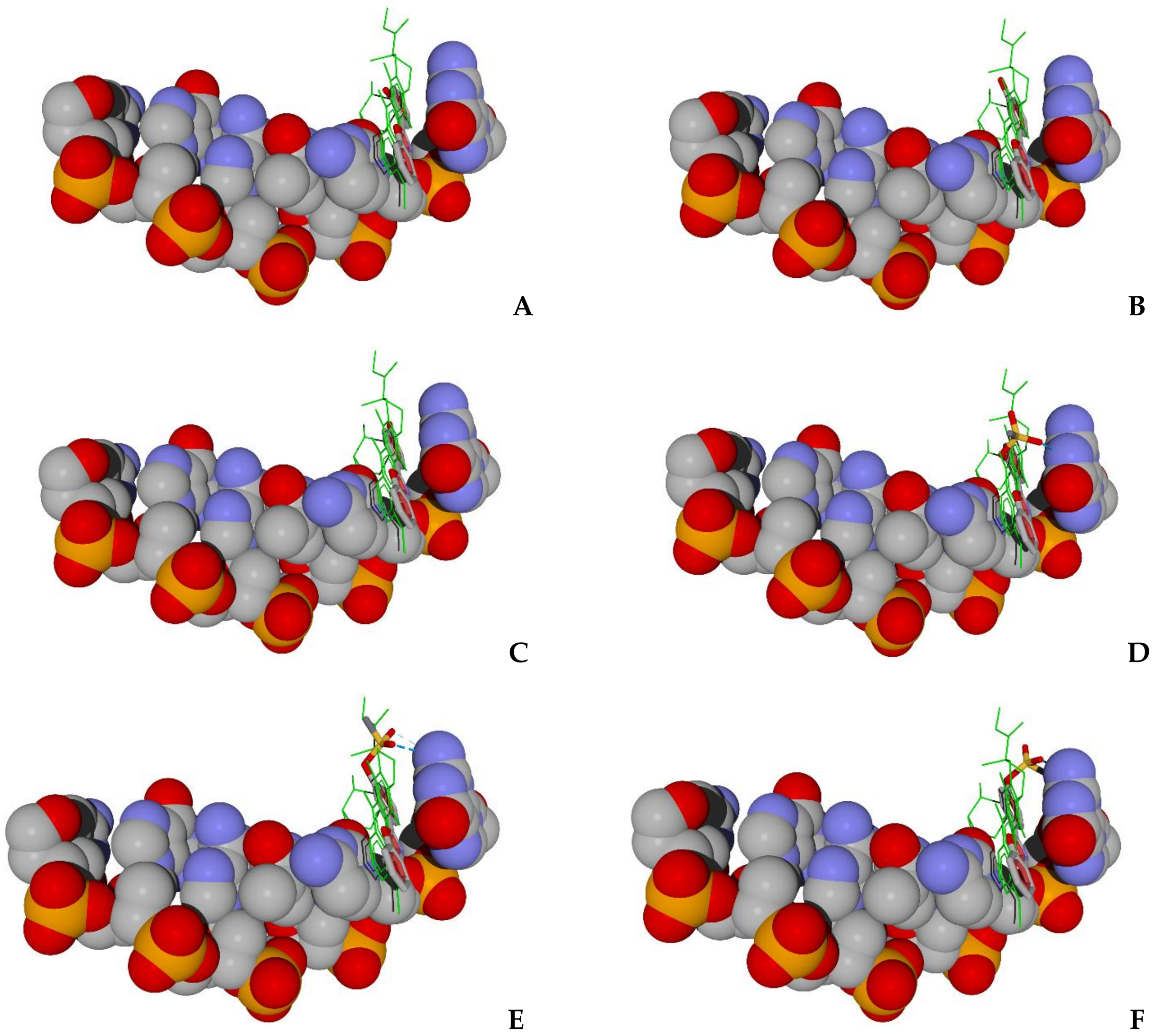

Figure S5. The docking poses (sticks) of compounds **BOo** (Panel A), **BOm** (Panel B), **BOp** (Panel C), **BOSo** (Panel D), **BOSm** (Panel E), and **BOSp** (Panel F) superimposed on the co-crystallized doxorubicin molecule (thin green sticks) in DNA strand (PDB: 151D). Hydrogen bonds are shown in dashed blue lines.

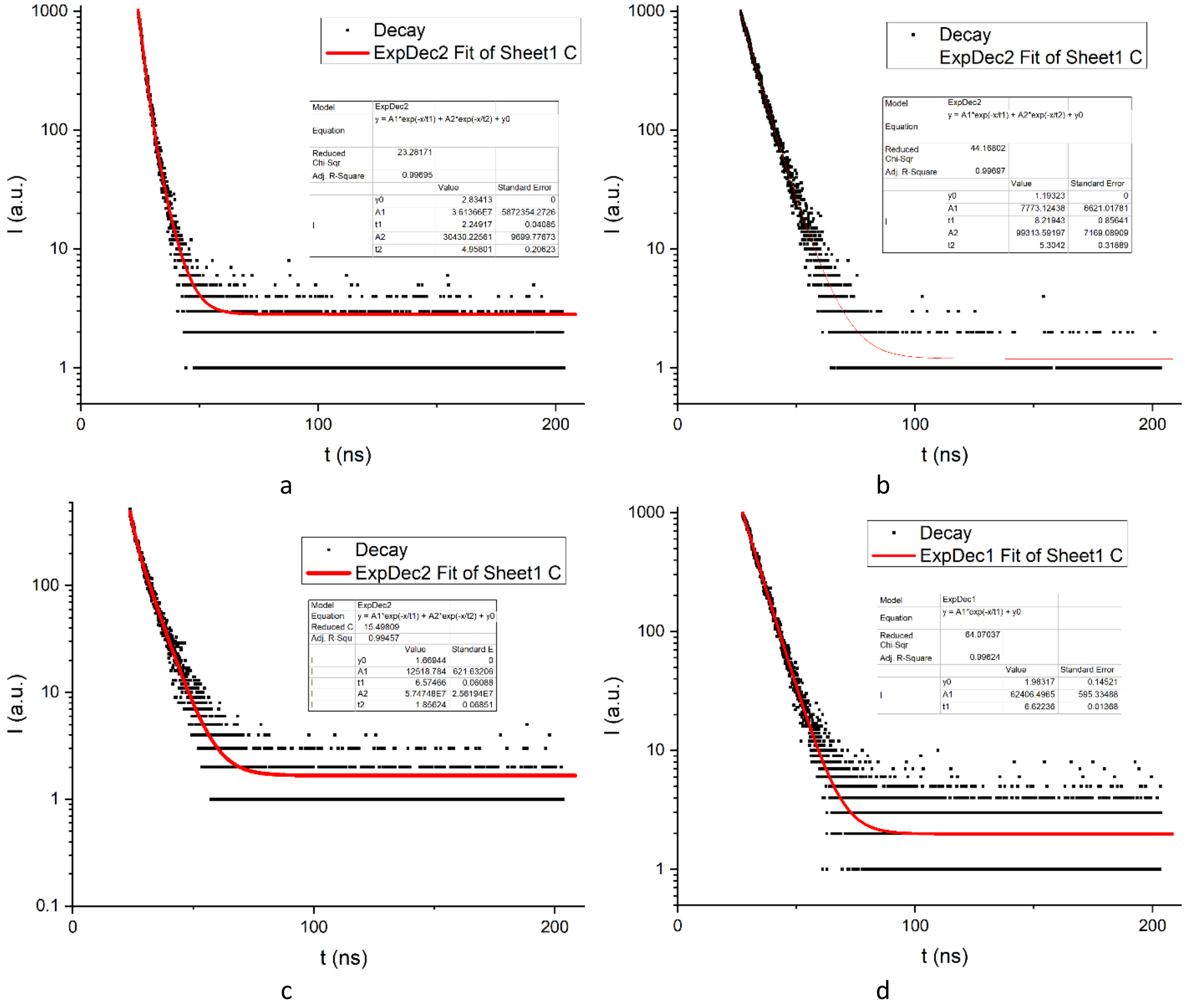

Figure S6. Emission decay plots in the solid state for compounds: (a) **BOo**; (b) **BOSp**; (c) **BOSm**; (d) **BOSo**.

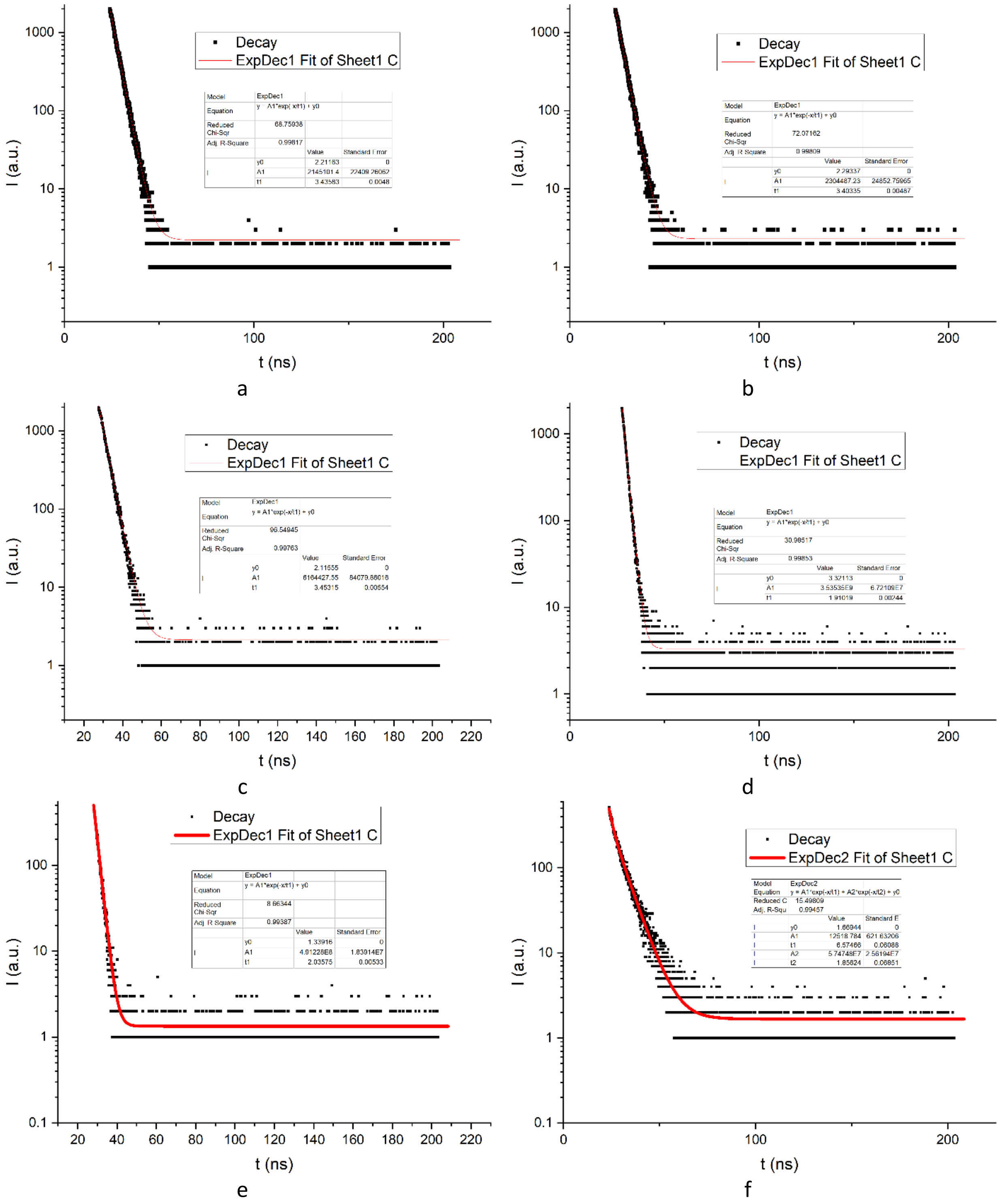

Figure S7. Emission decay plots in acetonitrile solution (C = 1.0·10^{-3} M) for compounds: (a) **BOo**; (b) **BOp**; (c) **BOm**; (d) **BOSp**; (e) **BOSm**; (f) **BOSo**.

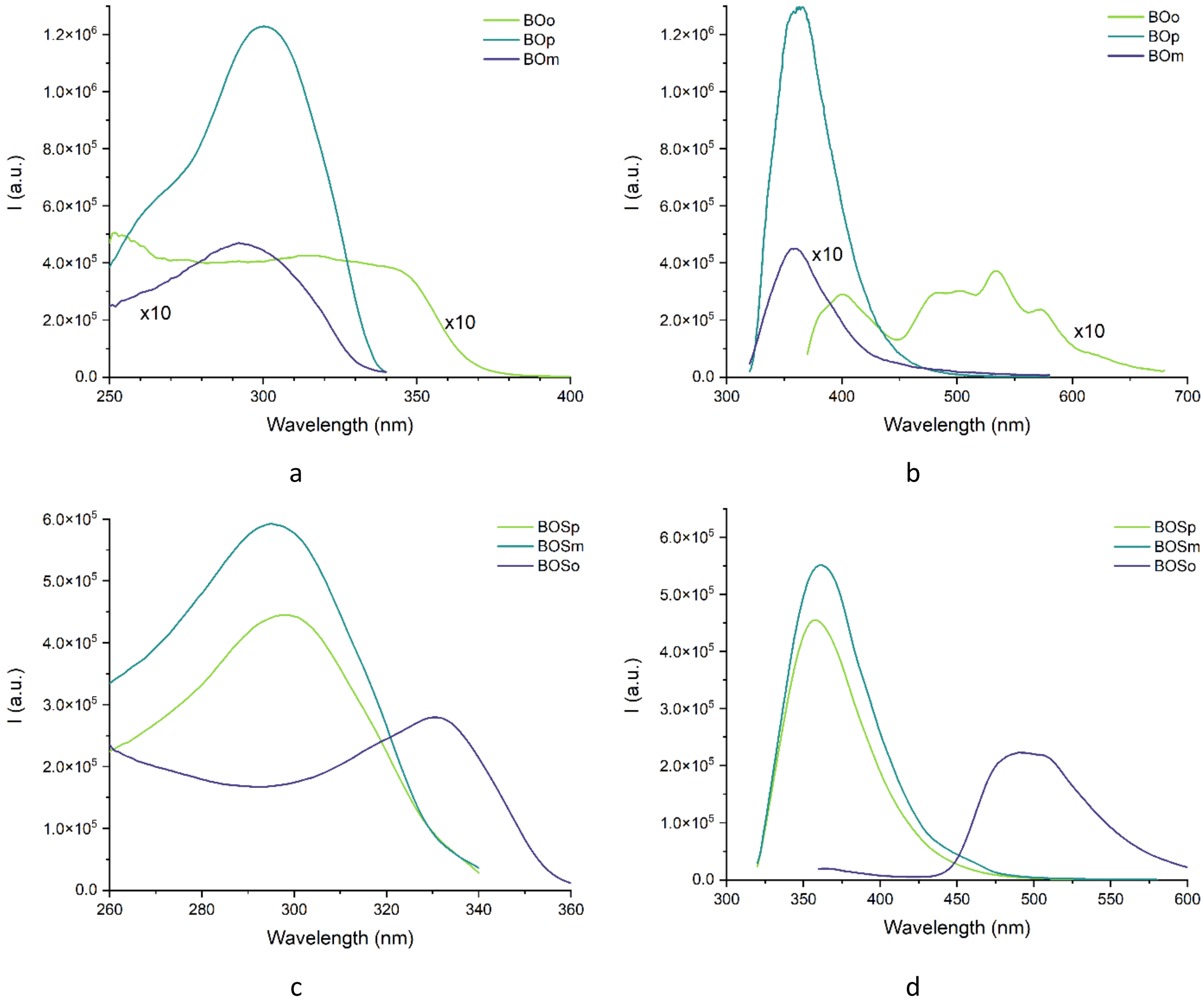

Figure S8. Excitation and emission spectra in phosphate buffer solution ($1.0 \cdot 10^{-3}$ M): (a) normalized excitation spectra of 2-(hydroxyphenyl)benzoxazoles, λ_{em} = 360 nm (**BOp**, **BOm**), 480 nm (**BOo**); (b) normalized emission spectra of 2-(hydroxyphenyl)benzoxazoles, λ_{ex} = 300 nm (**BOp**, **BOm**), 350 nm (**BOo**) (c) excitation spectra of 2-(fluorosulfatophenyl)benzoxazoles, λ_{em} = 360 nm (**BOSp**, **BOSm**), 500 nm (**BOSo**); (d) emission spectra of 2-(fluorosulfatophenyl)benzoxazoles, λ_{ex} = 300 nm (**BOSp**, **BOSm**), 340 nm (**BOSo**).

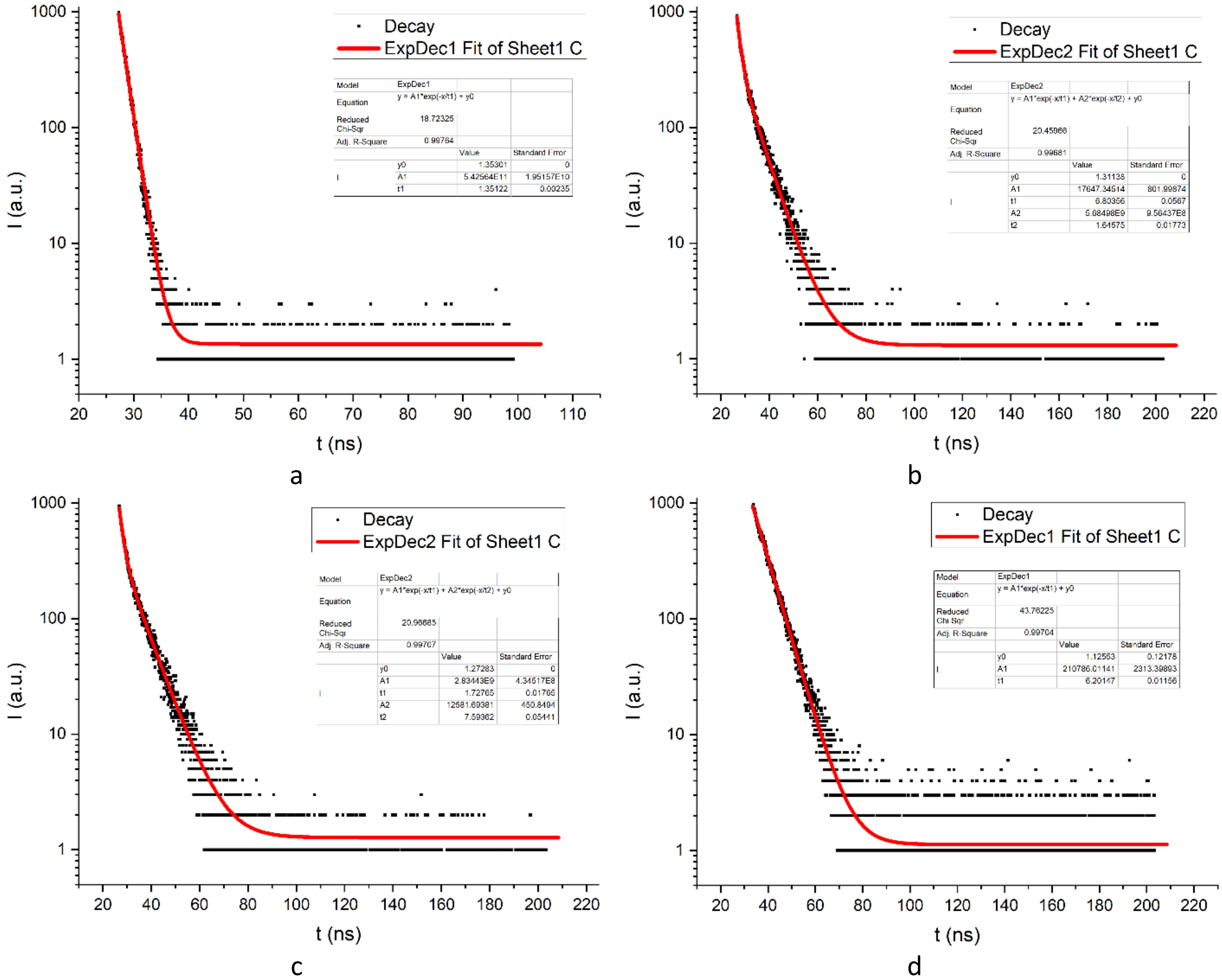

Figure S9. Emission decay plots in phosphate buffer solution (C = 1.0·10^{-3} M) for compounds: (a) **BOp**; (b) **BOSp**; (c) **BOSm**; (d) **BOSo**.

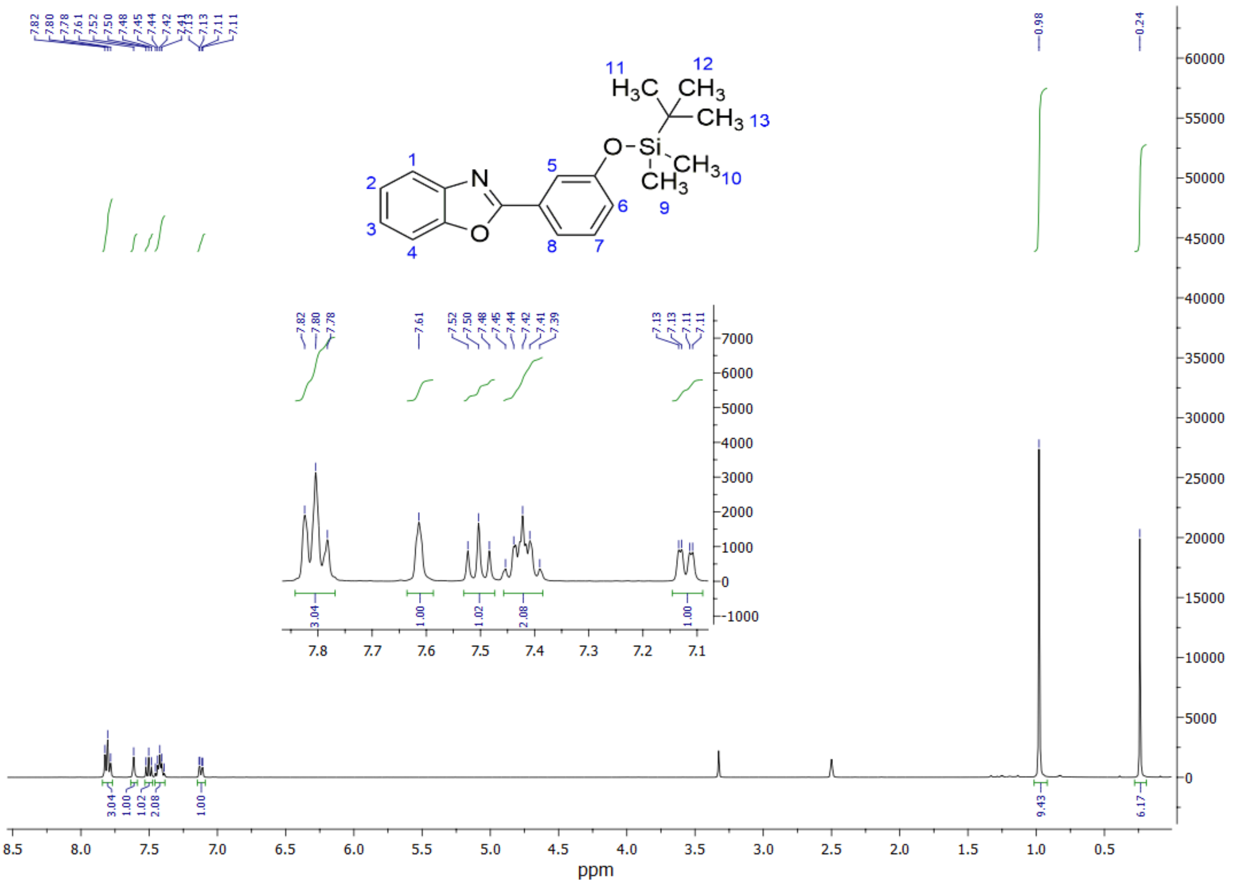

Figure S10. ^{1}H NMR spectrum of compound **BOmTBS** in DMSO-d6 (400 MHz).

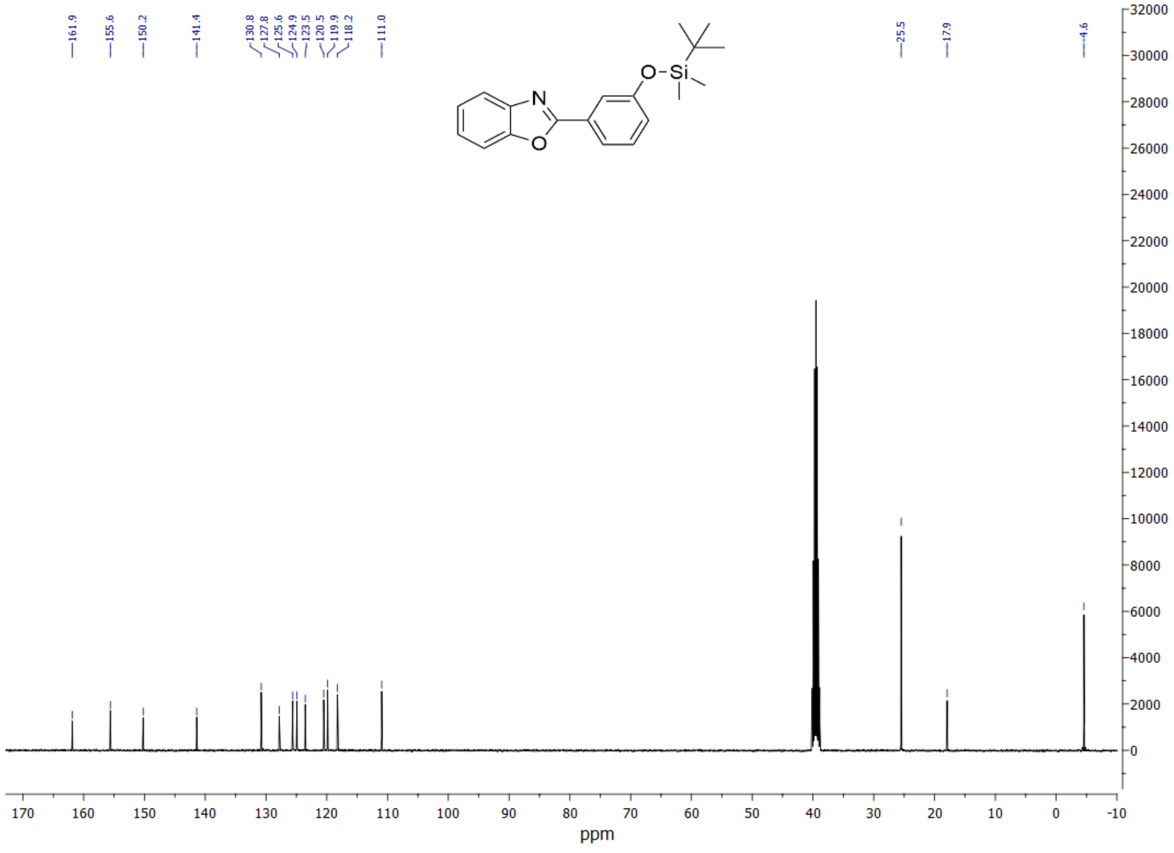

Figure S11. ^{13}C NMR spectrum of compound **BOmTBS** in DMSO-d6 (100 MHz).

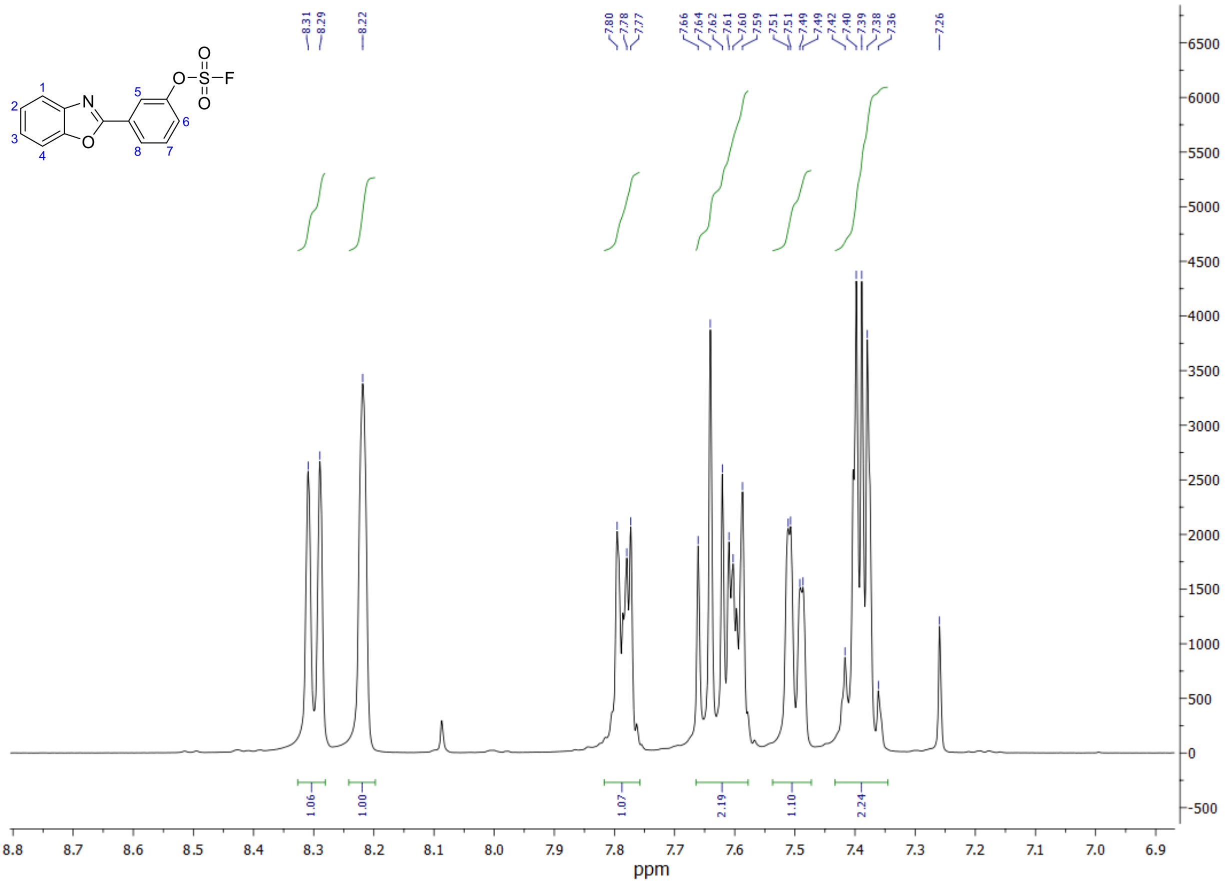

Figure S12. ^{1}H NMR spectrum of compound **BOSm** in $CDCl_3$ (400 MHz).

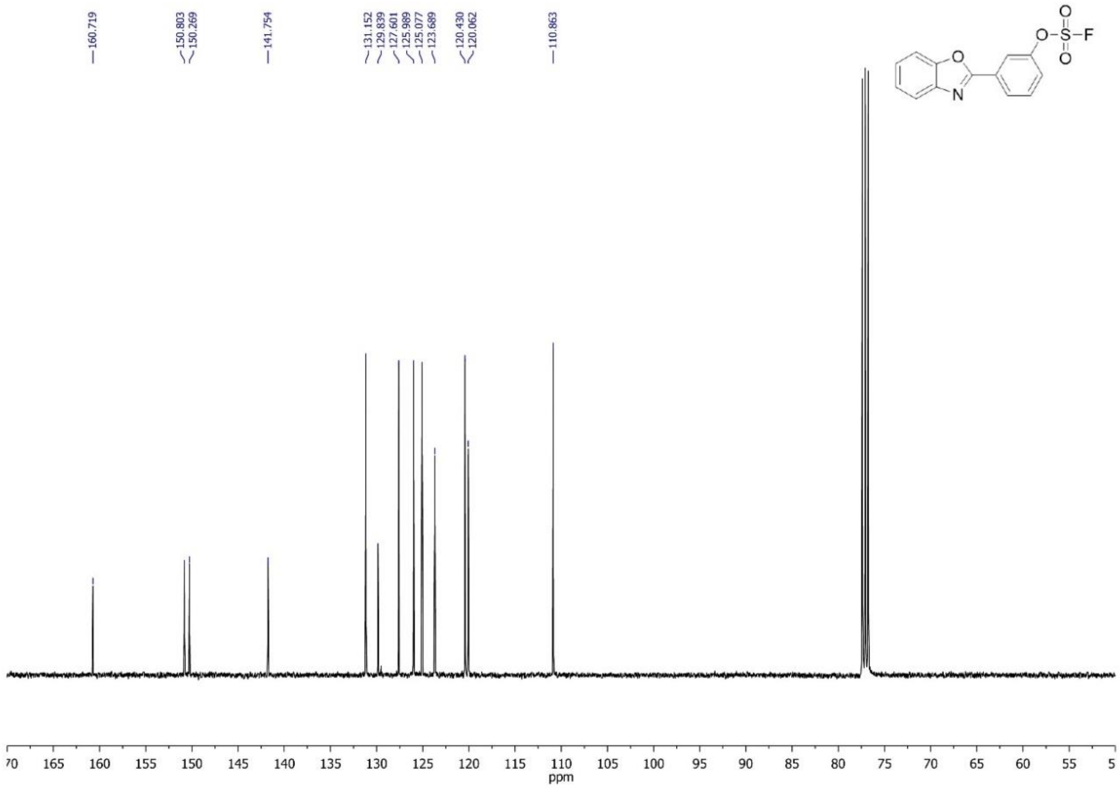

Figure S13. ^{13}C NMR spectrum of compound **BOSm** in $CDCl_3$ (100 MHz).

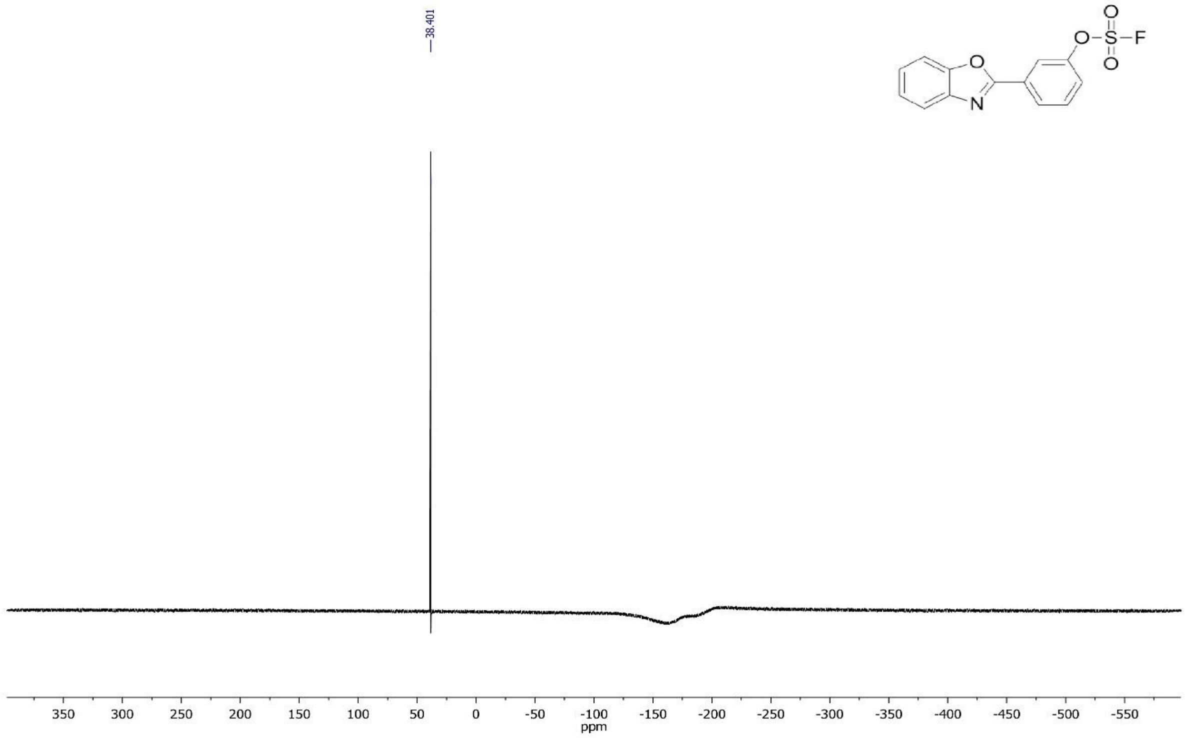

Figure S14. ^{19}F NMR spectrum of compound **BOSm** in $CDCl_3$ (376 MHz).

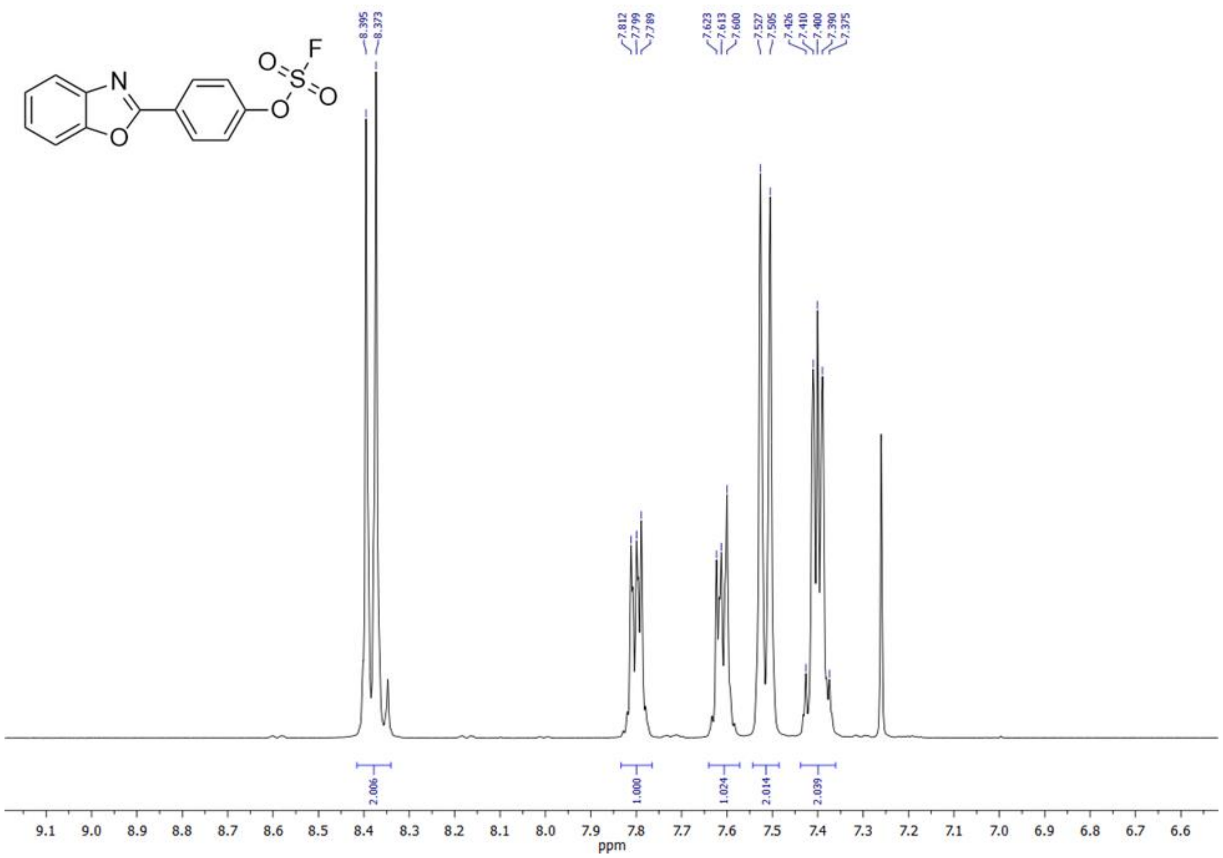

Figure S15. ^{1}H NMR spectrum of compound **BOSp** in $CDCl_3$ (400 MHz).

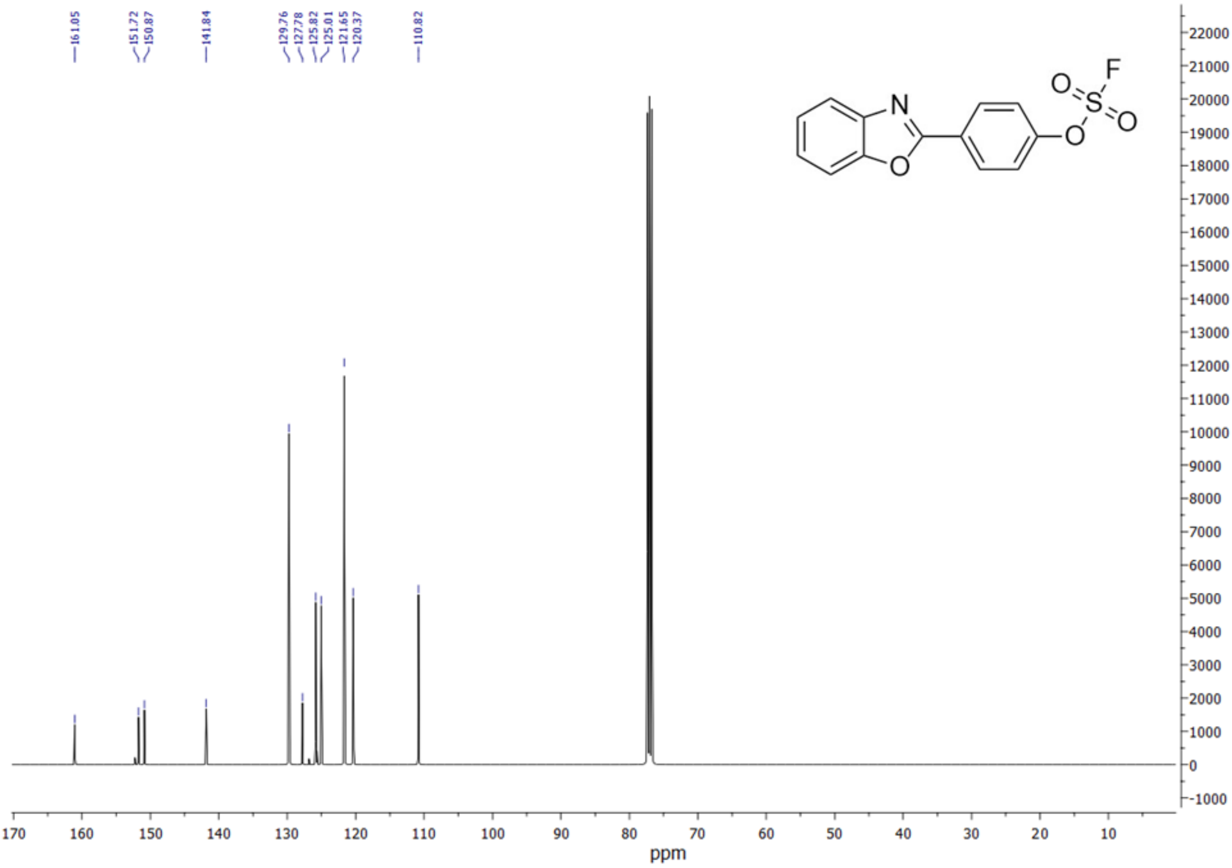

Figure S16. ^{13}C NMR spectrum of compound **BOSp** in $CDCl_3$ (100 MHz).

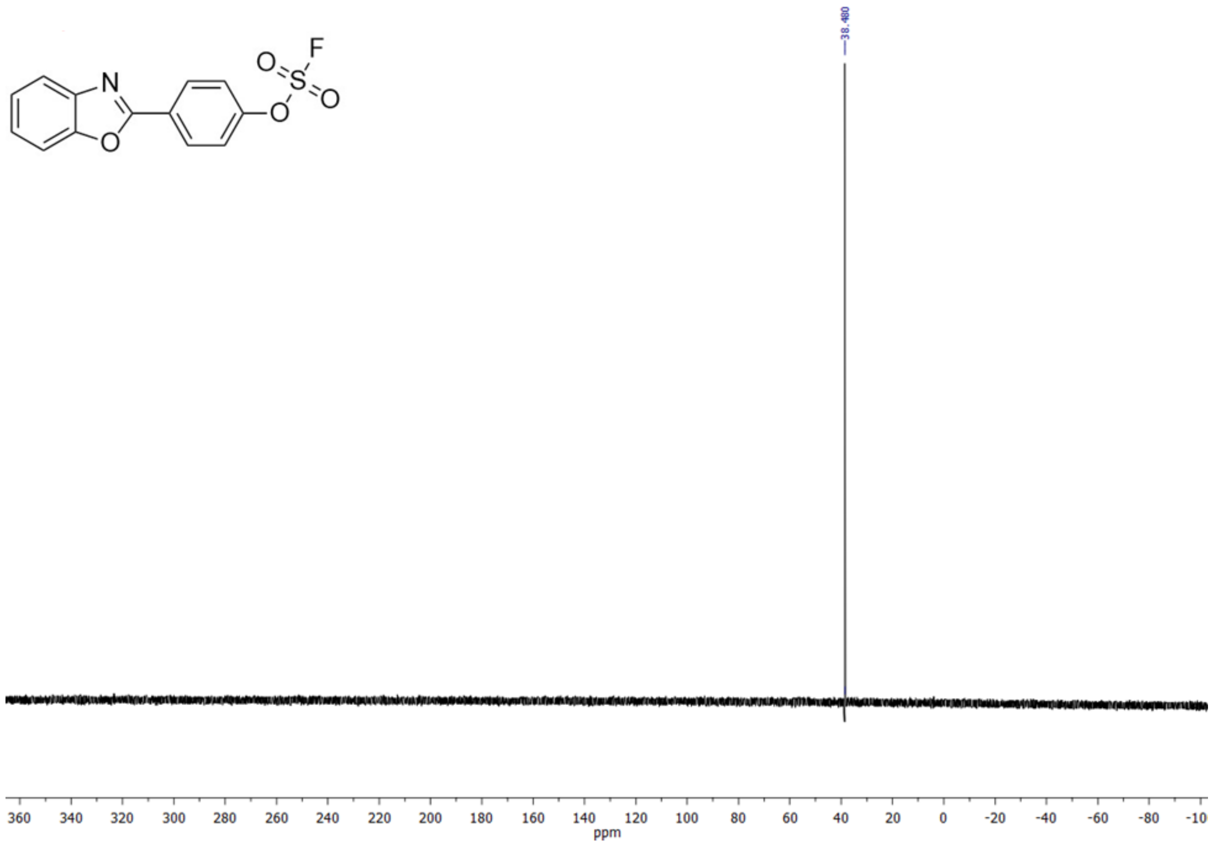

Figure S17. ^{19}F NMR spectrum of compound **BOSp** in $CDCl_3$ (376 MHz).

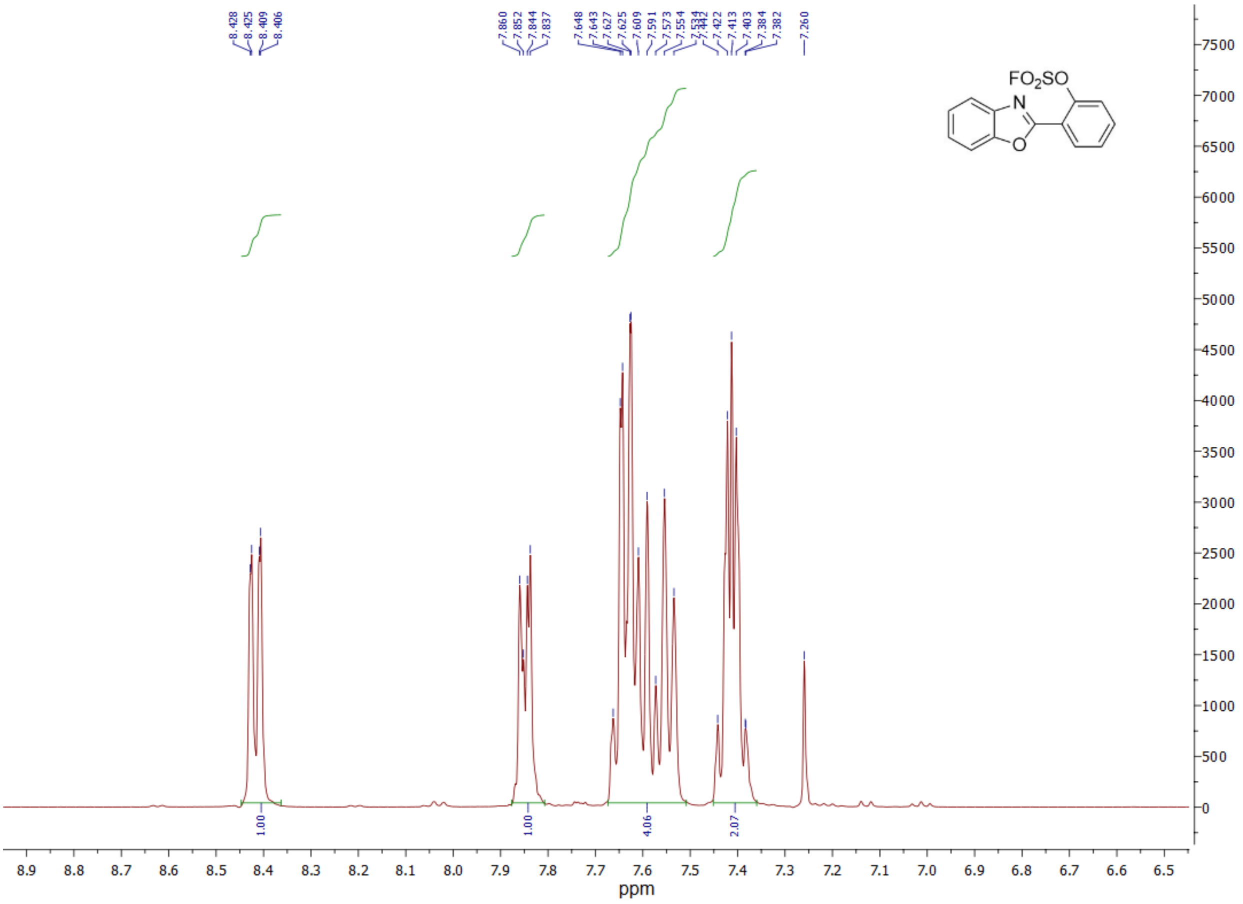

Figure S18. ^{1}H NMR spectrum of compound **BOSo** in $CDCl_3$ (400 MHz).

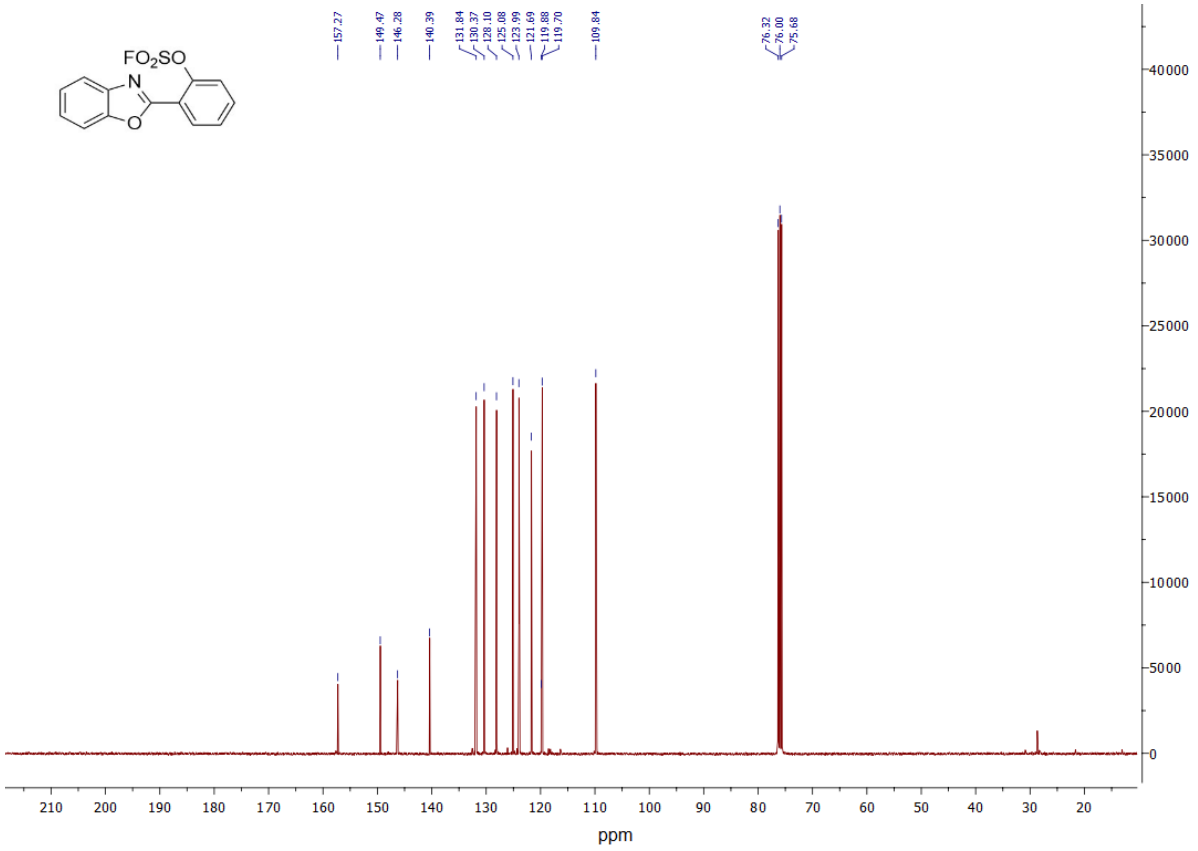

Figure S19. ^{13}C NMR spectrum of compound **BOSo** in $CDCl_3$ (100 MHz)**.**

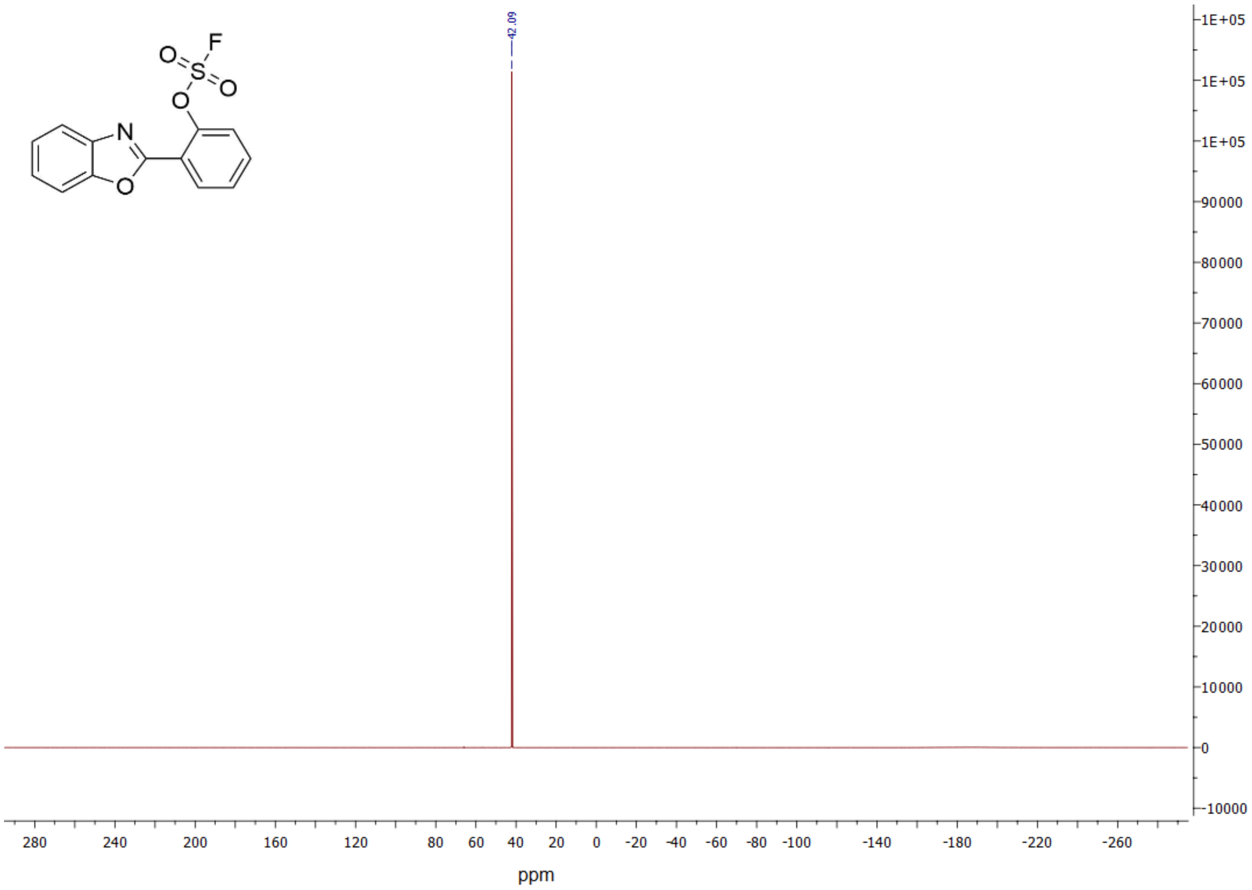

Figure S20. ^{19}F NMR spectrum of compound **BOSo** in $CDCl_3$ (376 MHz).

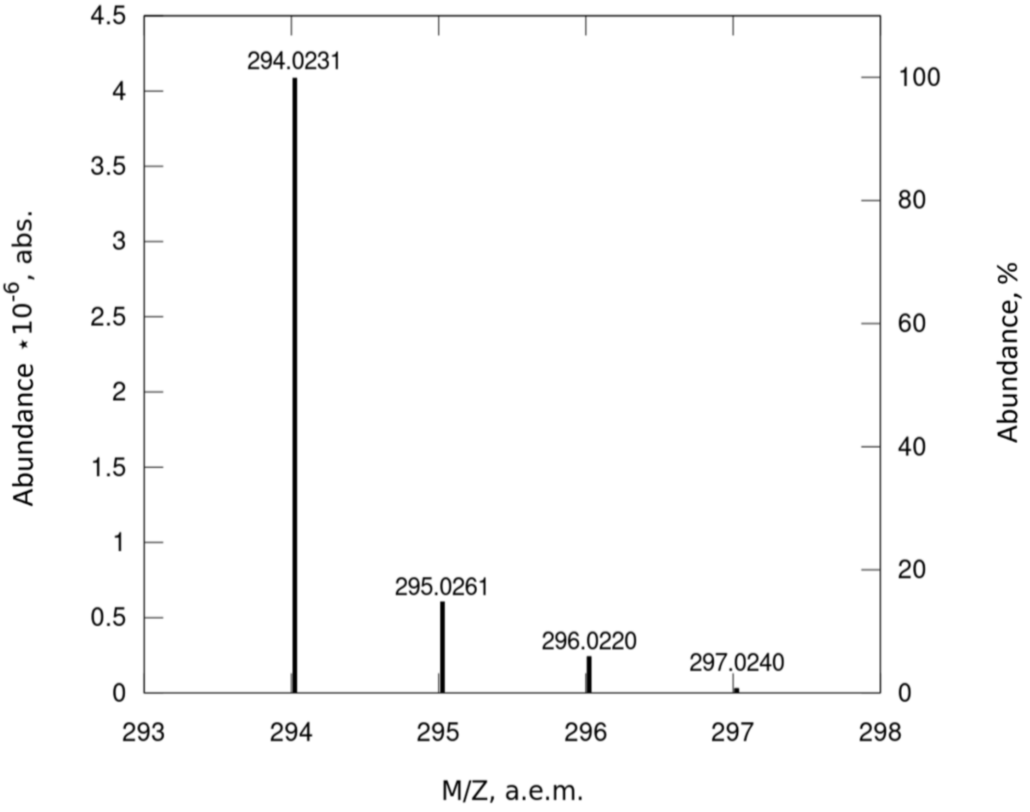

Figure S21. Peaks in the HRMS spectrum of Compound **BOSp**.

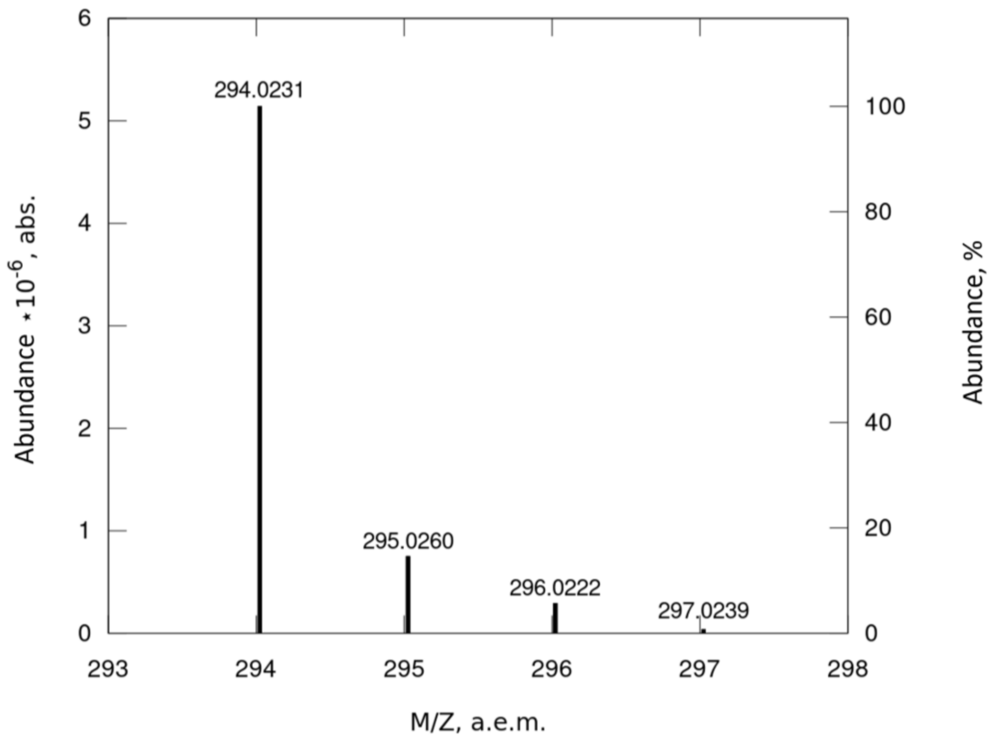

Figure S22. Peaks in the HRMS spectrum of Compound **BOSm**.

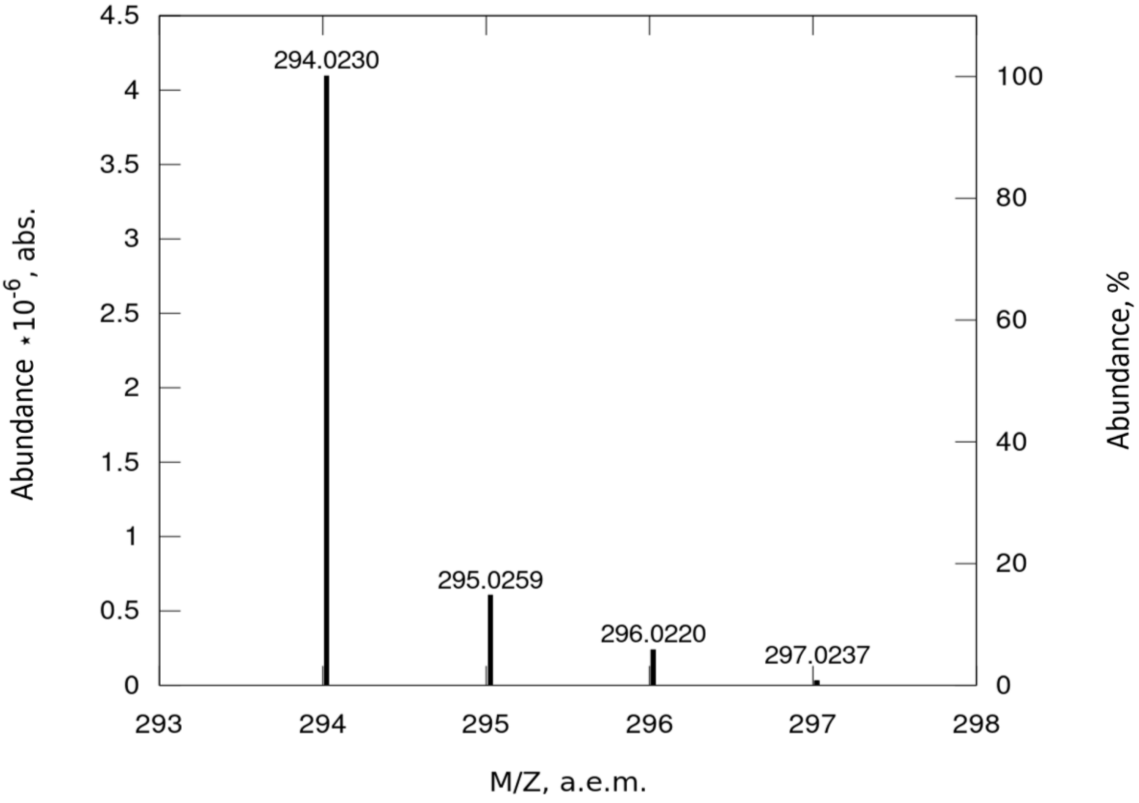

Figure S23. Peaks in the HRMS spectrum of Compound **BOSo**.
